# Evaluating Generative AI Large Language Models for Urticaria Management: A Comparative Analysis of DeepSeek‐R1 and ChatGPT‐4o

**DOI:** 10.1002/clt2.70113

**Published:** 2025-11-27

**Authors:** Mengyao Yang, Jingchen Liang, Luyue Zhang, Hongshan Liu, Ying Chen, Yawen Wang, Chunzhi Qi, Yuxin Ma, Ziyun Gao, Xinyue Zhang, Xinwu Niu, Xiaopeng Wang, Jianwen Ren, Jingyi Yuan, Weihui Zeng, Zhao Wang

**Affiliations:** ^1^ Department of Dermatology the Second Affiliated Hospital of Xi'an Jiaotong University Xi'an China

**Keywords:** AI, ChatGPT‐4o, DeepSeek‐R1, large language model, urticaria

## Abstract

**Introduction:**

Urticaria is a prevalent condition affecting a significant portion of the global population. Both dermatologists and patients require access to up‐to‐date and accurate information. Traditional search engines often fall short in meeting these needs. Despite the growing reliance on AI for medical inquiries, the accuracy and quality of AI‐generated remain understudied. This study aims to evaluate and compare the performance of two widely used AI models, ChatGPT‐4o and DeepSeek‐R1, in addressing urticaria‐related queries.

**Methods:**

An e‐Delphi procedure was employed to generate and refine a set of urticaria‐related questions, as well as to develop an evaluation framework for AI‐generated responses. ChatGPT‐4o and DeepSeek‐R1 were then prompted with the finalized questions, and their responses were recorded. A single‐blind comparative assessment was conducted among 67 participants (29 dermatologists and 38 non‐dermatologists). The responses from both AI models were assessed across simplicity, accuracy, professionalism, clinical feasibility, comprehensibility, and completeness.

**Results:**

DeepSeek‐R1 outperformed ChatGPT‐4o in most metrics. Dermatologists rated DeepSeek significantly higher in simplicity (*p* < 0.001), accuracy (*p* < 0.001), completeness (*p* = 0.001), professionalism (*p* < 0.001), and clinical feasibility (*p* < 0.001). Non‐dermatologists found DeepSeek's responses more concise (*p* < 0.001) and comprehensible (*p* < 0.001). Both models showed comparable integration of cutting‐edge knowledge (*p* = 0.06), though DeepSeek exhibited greater output stability, as evidenced by lower standard deviations. When compared with the guidelines, the answers provided by DeepSeek‐R1 contained no errors, while ChatGPT‐4o made errors in three clinical questions.

**Conclusion:**

AI‐generated answers require rigorous evaluation to ensure their reliability and suitability for medical applications. Based on the current study, DeepSeek‐R1 outperforms ChatGPT‐4o in addressing urticaria‐related queries, demonstrating higher potential for both clinical and patient use.

## 1 Introduction

Urticaria is characterized by pruritic wheals, affects approximately 20% of the global population at some point in their lives. Among these cases, 20%–45% develop chronic urticaria, which persists for more than six weeks [1, 2, 3]. While acute urticaria is commonly associated with allergies, chronic urticaria has a more diverse and complex pathogenesis [4, 5]. Various treatments have been explored for chronic urticaria, including antihistamines and anti‐IgE antibodies. Emerging therapies, such as small‐molecule Bruton's tyrosine kinase inhibitors, Janus kinase inhibitors, and anti‐c‐KIT antibodies, are also being investigated [6, 7]. The complex pathogenesis, chronic course, and resistance to treatment increase the need for up‐to‐date information on urticaria for both physicians and patients. Physicians require access to the latest research and clinical guidelines, while patients seek clear and easily understandable explanations of symptoms, treatment options, and prognosis. However, misinformation or overly technical language in search results can lead to delayed care, increased patient anxiety, or inappropriate self‐treatment.

Traditional search engines are effective for general inquiries but often struggle to synthesize specialized knowledge for professional use. In recent years, artificial intelligence (AI)‐powered searching tools have been widely adopted in various fields. AI‐driven search tools have improved the speed and accuracy of information retrieval. Advanced large language models (LLMs) have emerged as essential tools for processing and delivering specialized medical information [8].

Among the most prominent AI models, ChatGPT (developed by OpenAI) and DeepSeek (developed by DeepSeek Inc.) are widely used and represent distinct paradigms in information retrieval. ChatGPT, based on OpenAI's GPT‐4o and its predecessors, is designed to generate human‐like text responses. GPT‐4o was built upon earlier versions that had more limited reasoning capabilities. It was specifically trained to understand and generate natural language for tasks such as answering questions, providing explanations, generating content, and engaging in dialog [9]. DeepSeek, developed by DeepSeek Inc., is designed to process and analyze large datasets, extract insights, and provide actionable recommendations [10]. DeepSeek‐R1 is a model trained using reinforcement learning to generate “thinking‐aloud” tokens that reflect intermediate reasoning steps before producing a final answer to the user's query.

Several differences lie in architectural design, training data scope, and response‐generation strategies of those two models. ChatGPT prioritizes accessibility and breadth, while DeepSeek emphasizes accuracy and depth in specialized contexts. The ability of AI models to provide accurate and comprehensible answers directly impacts clinical decision‐making and public health outcomes. These divergences highlight the need to evaluate the capabilities of mainstream clinically available models in addressing domain‐specific challenges, particularly in environments of medical practice.

This study aims to compare the performance of ChatGPT and DeepSeek in addressing urticaria‐related queries for both dermatologists and individuals without a medical background, evaluating their technical accuracy and user‐centric clarity. Using the most commonly asked questions in clinical practice when treating urticaria, we compiled a list of queries and assessed the responses from ChatGPT‐4o and DeepSeek‐R1 based on their adherence to current guidelines, ease of understanding, and practical utility, etc. By analyzing the differences between the two models' outputs, we demonstrate that DeepSeek‐R1 outperformed ChatGPT‐4o in most metrics, including simplicity, accuracy, professionalism, feasibility, and comprehensibility. Our findings provide insight into the capabilities of ChatGPT‐4o and DeepSeek‐R1 in medical applications, particularly in addressing questions related to urticaria—one of the most prevalent dermatological conditions. Additionally, we highlight opportunities to optimize LLMs to better serve diverse audiences, from experts seeking in‐depth academic explanations to patients looking for clear, plain‐language answers.

## 2 Methods

### 2.1 Study Design

This was a single‐blind, cross‐sectional, comparative study conducted from February to March 2025, aimed at evaluating the performance of two large language models (LLMs)—ChatGPT‐4o and DeepSeek‐R1—in generating responses to urticaria‐related questions. The queries to both ChatGPT‐4o and DeepSeek‐R1 were conducted on February 9, 2025.

Participants included both dermatologists and non‐dermatologists. Dermatologist participants were recruited from our dermatology department and were all actively involved in the clinical diagnosis and treatment of urticaria. The non‐dermatologist group comprised individuals randomly selected from among patients visiting our department and healthy volunteers recruited through open invitations. Each evaluator sequentially assessed the responses of both models, with every individual providing scores for both models.

A modified electronic Delphi (eDelphi) process was employed to guide both the development of the urticaria‐related questions and the multi‐dimensional evaluation of the AI‐generated responses [11]. The entire study was conducted in Chinese, simulating real‐world interactions for native Chinese speakers seeking health‐related information through AI tools.

### 2.2 Question Design

An initial pool of 20 pre‐selected urticaria‐related questions was developed based on clinical guidelines and common patient concerns. These questions were reviewed by a panel of dermatologists using a Likert scale [12] ranging from “Strongly Disagree” to “Strongly Agree” (scored 1–5). Responses were collected and analyzed by the study sponsor (see Table S1). Based on statistical feedback, the questionnaire was modified and reevaluated in subsequent rounds. This iterative process continued until all dermatologists rated each item as “Agree” or “Strongly Agree”. Ultimately, 12 finalized questions were selected for use in this study. The finalized questions were then submitted to ChatGPT‐4o and DeepSeek‐R1, both queried in Chinese. AI‐generated responses were collected and anonymized for subsequent evaluation.

### 2.3 Evaluation Process and Scoring Criteria

A panel of dermatologists and non‐dermatologists was recruited to independently evaluate the AI‐generated responses. Evaluation dimensions were tailored to the expertise of each group. Dermatologists assessed each response across six metrics: simplicity, accuracy, completeness, professionalism, degree of cutting‐edge knowledge integration, and feasibility for clinical practice. Non‐dermatologists evaluated responses based on their accessibility to a general audience, focusing specifically on accuracy and comprehensibility.

Each evaluator was blinded to the source of the responses (ChatGPT‐4o or DeepSeek‐R1) and independently reviewed the answers to the 12 final questions. All responses were randomized and de‐identified to minimize bias. Evaluators used a 4‐level rating scale to score each response (0 = Awful; 1 = Normal; 2 = Good; 3 = Excellent). Scores were recorded for each question and evaluations from dermatologists and non‐dermatologists were analyzed separately. Finally, comparative analyses were conducted across models (ChatGPT‐4o vs. DeepSeek‐R1) allowing for a comprehensive evaluation of each model's ability to generate clinically sound and accessible content.

### 2.4 Statistical Analysis

Statistical analyses were performed by Statistical Package for Social Science (SPSS) statistical program version (23.0). This study employed non‐normally distributed paired samples, hence the Wilcoxon matched‐pairs signed‐rank test was applied to compare rank‐based data. *p* < 0.05 was considered statistically significant.

## 3 Results

### 3.1 Demographic Characteristics of Evaluators

A total of 67 individuals completed evaluations. Among these, 29 were dermatologists, and 38 were non‐dermatologists. The gender distribution included 16 males (23.88%) and 51 females (76.12%). In terms of age, the largest proportion of participants were 26–45 years old (30 individuals, 44.78%), followed by those aged 18–25 years old (29 individuals, 43.28%), 6 participants (8.96%) were aged 45–60 years, 3 (4.48%) were under 18 years old, and 1 (1.49%) was over 60 years old. Detailed demographic data are summarized in Table 1.

**TABLE 1:** Participants demographic and characteristics.

Characteristic	Value		
	Dermatologist	Non‐dermatologist	Total
Gender			
Male, *n* (%)	5 (7.46%)	11 (16.42%)	16 (23.88%)
Female, *n* (%)	24 (35.82%)	27 (40.30%)	51 (76.12%)
Age			
< 18	0	3 (4.48%)	3 (4.48%)
18–25	17 (25.37%)	12 (17.91%)	29 (43.28%)
26–45	11 (16.42%)	19 (28.36%)	30 (44.78%)
45–60	2 (2.99%)	4 (5.97%)	6 (8.96%)
> 60	0	1 (1.49%)	1 (1.49%)

### 3.2 Overall Performance Comparison of the Two LLMs

**FIGURE 1:**
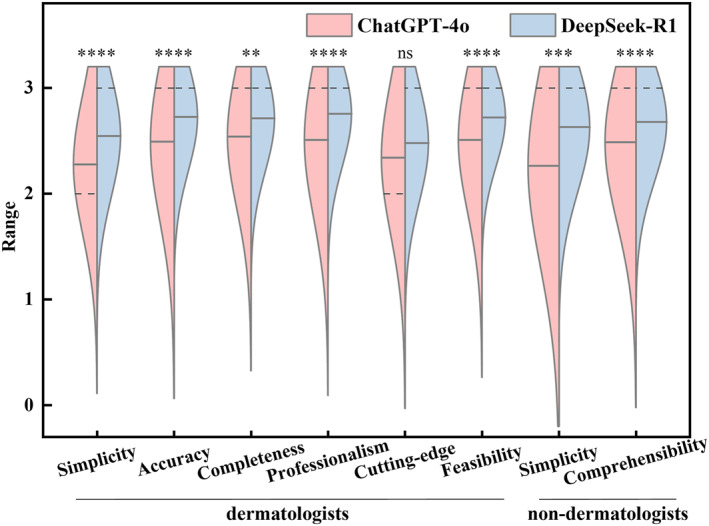
Comparison of score distribution between DeepSeek‐R1 and ChatGPT‐4o across evaluation domains. The responses generated by DeepSeek‐R1 and ChatGPT‐4o were evaluated by 29 dermatologists and 38 non‐dermatologists. Dermatologists assessed each answer based on six criteria: simplicity, accuracy, completeness, professionalism, integration of cutting‐edge knowledge, and clinical feasibility. Non‐dermatologists evaluated responses for accuracy and comprehensibility to a general audience. Solid lines represent the peak value (mean), dashed lines represent the median with *n* = 67. The *p* values were obtained by comparing between groups. ns *p* > 0.05, **p < 0.01.***p < 0.001. ****p < 0.0001.

The average and median scores of ChatGPT‐4o and DeepSeek‐R1 across all evaluation metrics fall between 2 and 3, corresponding to the “Good” to “Excellent” rating (Figure 1). The Figure 1 showed their distributions were closely aligned, with averages predominantly clustered around 2 and 3. Differences in distribution revealed that across all metrics, DeepSeek‐R1's scores were more concentrated in the high‐score range compared to ChatGPT‐4o. ChatGPT‐4o′s average scores (solid line) were consistently lower than DeepSeek‐R1's, with significant differences observed in 7 metrics. The largest gap was observed in simplicity ratings by non‐dermatologists (*p* < 0.001), while the smallest gap was in cutting‐edge (*p* = 0.06). Regarding medians (dashed line), only simplicity (2 vs. 3) and cutting‐edge (2 vs. 3) showed differences, while the median for all other indicators were 3.

### 3.3 Comparison of ChatGPT‐4o and DeepSeek‐R1's Answers by Individual Question Analysis

In the present study, 12 distinct questions were generated, and the answers provided by the two AI models, along with participant ratings, were recorded in Table 2. Overall, DeepSeek's responses were more concise than ChatGPT's across all 12 questions, with question 4, 5, 8, and 12 showing a statistically significant difference in simplicity by dermatologists or non‐dermatologists. DeepSeek's answers were also completer and more detailed, addressing aspects that were lacking in ChatGPT's responses. For example, DeepSeek provided a clearer classification of chronic spontaneous and inducible urticaria in question 2, a more detailed explanation of the pathogenesis and target pathways of urticaria in question 3, and a specific scale for urticaria in question 7. Additionally, DeepSeek individually addressed different types of urticaria in question 8 and discussed the long‐term use of antihistamines in different populations in question 11. These findings highlight DeepSeek's ability to deliver more comprehensive and precise responses compared to ChatGPT.

**TABLE 2:** Comparasion of ChatGPT‐4o's and DeepSeek‐R1's amswers about urticaria.

				ChatGPT‐4o	DeepSeek‐R1	*p*		
Q1. What is urticaria?	Response summary			Contained a definition, main symptoms, common triggers, classification, treatments, and an explanation of wheal in 551 words.	Answers included definition, clinical presentation, pathogenesis, classification, and treatment in 399 words.			
	Evaluation	Dermatologist	Simplicity	2.00 (2.00–3.00)	2.00 (2.00–3.00)	0.41	0.009^bb^	0.006**
		Accuracy	3.00 (2.00–3.00)	3.00 (2.00–3.00)	0.60			
		Completeness	3.00 (2.00–3.00)	3.00 (2.00–3.00)	0.66			
		Professionalism	3.00 (2.00–3.00)	3.00 (2.00–3.00)	0.15			
		Cutting‐edge	2.00 (2.00–3.00)	2.00 (2.00–3.00)	0.11			
		Feasibility	3.00 (2.00–3.00)	3.00 (2.00–3.00)	0.12			
	Non‐dermatologist	Simplicity	3.00 (2.00–3.00)	3.00 (2.00–3.00)	0.33	0.30		
		Comprehensibility	3.00 (2.00–3.00)	3.00 (2.75–3.00)	0.04^a^			
Q2. How is urticaria classified?	Response summary			It divided urticaria into four categories: acute, chronic, special types, accompanied by other symptoms, without mentioning spontaneous urticaria and induced urticaria in 922 words.	It categorized urticaria in terms of duration, predisposing factors, pathomechanisms, and specific types, respectively, and was more comprehensive, totaling 701 words.			
	Evaluation	Dermatologist	Simplicity	2.00 (2.00–3.00)	2.00 (2.00–3.00)	0.30	0.001^bb^	< 0.001***
			Accuracy	3.00 (2.00–3.00)	3.00 (2.50–3.00)	0.20		
			Completeness	3.00 (2.00–3.00)	3.00 (2.00–3.00)	0.43		
			Professionalism	3.00 (2.00–3.00)	3.00 (3.00–3.00)	0.09		
			Cutting‐edge	2.00 (2.00–3.00)	3.00 (2.00–3.00)	0.09		
			Feasibility	2.00 (2.00–3.00)	3.00 (2.00–3.00)	0.12		
		Non‐dermatologist	Simplicity	3.00 (1.75–3.00)	3.00 (2.00–3.00)	0.27	0.17	
			Comprehensibility	3.00 (2.00–3.00)	3.00 (2.75–3.00)	0.38		
Q3. What causes urticaria?	Response summary			It mentioned allergic reactions, environmental factors, endocrine, autoimmune, genetic factors and other predisposing factors, but did not explain the specific mechanisms, totaling 574 words.	It discussed the specific pathogenesis and common triggers of urticaria, respectively, and details how urticaria developed in 1098 words.			
	Evaluation	Dermatologist	Simplicity	2.00 (2.00–3.00)	3.00 (2.00–3.00)	0.12	< 0.001^bbb^	0.007**
			Accuracy	3.00 (2.00–3.00)	3.00 (2.00–3.00)	0.49		
			Completeness	3.00 (2.00–3.00)	3.00 (2.50–3.00)	0.15		
			Professionalism	3.00 (2.00–3.00)	3.00 (3.00–3.00)	0.14		
			Cutting‐edge	2.00 (2.00–3.00)	3.00 (2.00–3.00)	0.02^a^		
			Feasibility	3.00 (2.00–3.00)	3.00 (3.00–3.00)	0.02^a^		
		Non‐dermatologist	Simplicity	3.00 (2.00–3.00)	3.00 (2.00–3.00)	0.72	0.31	
			Comprehensibility	3.00 (2.00–3.00)	3.00 (2.00–3.00)	0.27		
Q4. How is urticaria diagnosed?	Response summary			The answer included a history taking, clinical signs, laboratory tests such as allergen testing, and differential diagnosis in 1154 words.	Similar to the ChatGPT's response, it also included history taking, physical examination, laboratory tests, and differential diagnosis in 464 words.			
	Evaluation	Dermatologist	Simplicity	2.00 (2.00–3.00)	3.00 (2.00–3.00)	0.007^aa^	0.004^bb^	< 0.001***
			Accuracy	3.00 (2.00–3.00)	3.00 (3.00–3.00)	0.08		
			Completeness	3.00 (2.00–3.00)	3.00 (2.00–3.00)	0.48		
			Professionalism	3.00 (2.00–3.00)	3.00 (3.00–3.00)	0.10		
			Cutting‐edge	2.00 (2.00–3.00)	2.00 (2.00–3.00)	0.81		
			Feasibility	3.00 (2.00–3.00)	3.00 (2.00–3.00)	0.37		
		Non‐dermatologist	Simplicity	3.00 (1.00–3.00)	3.00 (2.00–3.00)	0.07	0.08	
			Comprehensibility	3.00 (2.00–3.00)	3.00 (2.00–3.00)	0.50		
Q5. Are laboratory tests necessary for diagnosing acute urticaria?	Response summary			Common laboratory tests and the appropiate timing were discussed separately, concluding that “Laboratory tests are not usually needed, but may be recommended depending on the condition” in 696 words.	Similar to the ChatGPT's answer, it also discussed the tests that need to be refined when infection, allergies, and other diseases were suspected, totaling 309 words.			
	Evaluation	Dermatologist	Simplicity	2.00 (2.00–3.00)	3.00 (2.00–3.00)	0.03^a^	0.04^b^	0.003**
			Accuracy	3.00 (2.00–3.00)	3.00 (2.00–3.00)	0.48		
			Completeness	3.00 (2.00–3.00)	3.00 (2.50–3.00)	0.43		
			Professionalism	3.00 (2.00–3.00)	3.00 (2.00–3.00)	0.73		
			Cutting‐edge	2.00 (2.00–3.00)	3.00 (2.00–3.00)	0.64		
			Feasibility	3.00 (2.00–3.00)	3.00 (2.50–3.00)	0.21		
		Non‐dermatologist	Simplicity	3.00 (2.00–3.00)	3.00 (2.00–3.00)	0.04^a^	0.03^c^	
			Comprehensibility	3.00 (2.00–3.00)	3.00 (2.00–3.00)	0.31		
Q6. What are the causes of chronic spontaneous urticaria, and how is it classified?	Response summary			The response contradicted the established clinical guidelines by categorizing chronic spontaneous urticaria into idiopathic and secondary forms, while failing to explain its pathogenesis, and was presented in 1096 words.	DeepSeek's response accurately addressed the pathogenesis of CSU without any notable contradictions to established clinical guidelines and was more concise, totaling 309 words.			
	Evaluation	Dermatologist	Simplicity	2.00 (2.00–3.00)	2.00 (2.00–3.00)	0.60	0.07	0.008**
			Accuracy	3.00 (2.00–3.00)	3.00 (2.00–3.00)	0.10		
			Completeness	3.00 (2.00–3.00)	3.00 (3.00–3.00)	0.49		
			Professionalism	3.00 (2.00–3.00)	3.00 (2.00–3.00)	0.43		
			Cutting‐edge	3.00 (2.00–3.00)	3.00 (2.00–3.00)	0.82		
			Feasibility	3.00 (2.00–3.00)	3.00 (2.50–3.00)	0.21		
		Non‐dermatologist	Simplicity	3.00 (1.00–3.00)	3.00 (2.00–3.00)	0.05	0.04^c^	
			Comprehensibility	3.00 (2.00–3.00)	3.00 (2.00–3.00)	0.37		
Q7. How is the severity and activity of urticaria assessed?	Response summary			Symptoms, rash, concomitant symptoms, degree of itching, and drug reactions were mentioned, but none of the standardized scales were mentioned, for a total of 889 words.	Not only was the rash, degree of itching, frequency, and concomitant symptoms mentioned, but also a standardized rating scale and a comprehensive assessment in 588 words.			
	Evaluation	Dermatologist	Simplicity	2.00 (1.50–3.00)	2.00 (2.00–3.00)	0.14	< 0.001^bbb^	< 0.001***
			Accuracy	3.00 (2.00–3.00)	3.00 (2.50–3.00)	0.24		
			Completeness	3.00 (2.00–3.00)	3.00 (3.00–3.00)	0.07		
			Professionalism	3.00 (2.00–3.00)	3.00 (3.00–3.00)	0.08		
			Cutting‐edge	3.00 (2.00–3.00)	3.00 (2.00–3.00)	0.18		
			Feasibility	3.00 (2.00–3.00)	3.00 (3.00–3.00)	0.06		
		Non‐dermatologist	Simplicity	2.50 (1.75–3.00)	3.00 (3.00–3.00)	0.005^aa^	0.003^cc^	
			Comprehensibility	3.00 (2.00–3.00)	3.00 (2.00–3.00)	0.18		
Q8. Is urticaria curable?	Response summary			It answered that question in terms of acute urticaria and chronic urticaria respectively and mentions treatments in 502 words.	Separate answers on the likelihood of curing acute urticaria, CSU, physical urticaria, and other urticaria, as well as treatments, totaling 401 words.			
	Evaluation	Dermatologist	Simplicity	3.00 (2.00–3.00)	3.00 (3.00–3.00)	0.04^a^	< 0.001^bbb^	< 0.001***
			Accuracy	3.00 (2.00–3.00)	3.00 (3.00–3.00)	0.07		
			Completeness	3.00 (2.00–3.00)	3.00 (3.00–3.00)	0.07		
			Professionalism	3.00 (2.00–3.00)	3.00 (3.00–3.00)	0.26		
			Cutting‐edge	2.00 (2.00–3.00)	3.00 (2.00–3.00)	0.29		
			Feasibility	3.00 (2.00–3.00)	3.00 (2.50–3.00)	0.71		
		Non‐dermatologist	Simplicity	3.00 (2.00–3.00)	3.00 (2.75–3.00)	0.02^a^	0.02^c^	
			Comprehensibility	3.00 (2.00–3.00)	3.00 (2.00–3.00)	0.34		
Q9. How is urticaria treated?	Response summary			It included medication, avoidance of triggers, lifestyle, and complementary therapies, but there were contradictions to the established clinical guidelines: the example was given of omalizumab, an anti‐IL‐5 biologic, in 1128 words.	Included treatments for acute urticaria, CSU, physical urticaria, and other urticaria in 613 words.			
	Evaluation	Dermatologist	Simplicity	2.00 (2.00–3.00)	3.00 (2.00–3.00)	0.13	0.002^bb^	< 0.001***
			Accuracy	3.00 (2.00–3.00)	3.00 (3.00–3.00)	0.08		
			Completeness	3.00 (2.00–3.00)	3.00 (2.50–3.00)	0.21		
			Professionalism	3.00 (2.00–3.00)	3.00 (2.50–3.00)	0.04^a^		
			Cutting‐edge	2.00 (2.00–3.00)	2.00 (2.00–3.00)	0.57		
			Feasibility	2.50 (1.75–3.00)	3.00 (2.00–3.00)	0.35		
		Non‐dermatologist	Simplicity	2.00 (2.00–3.00)	3.00 (2.00–3.00)	0.008^aa^	0.002^cc^	
			Comprehensibility	3.00 (2.00–3.00)	3.00 (2.00–3.00)	0.11		
Q10. What is omalizumab, and is it safe?	Response summary			Including mechanism, mode of use, safety, side effects of omalizumab in 1059 words.	The answer was similar to ChatGPT, including definitions, safety, side effects, precautions, and 328 words.			
	Evaluation	Dermatologist	Simplicity	2.00 (2.00–3.00)	3.00 (2.00–3.00)	0.06	0.07	0.004**
			Accuracy	3.00 (2.00–3.00)	3.00 (3.00–3.00)	0.15		
			Completeness	3.00 (2.00–3.00)	3.00 (2.00–3.00)	0.46		
			Professionalism	3.00 (2.00–3.00)	3.00 (3.00–3.00)	0.11		
			Cutting‐edge	3.00 (2.00–3.00)	3.00 (2.00–3.00)	0.75		
			Feasibility	3.00 (2.00–3.00)	3.00 (2.00–3.00)	0.49		
		Non‐dermatologist	Simplicity	3.00 (2.00–3.00)	3.00 (2.75–3.00)	0.03^a^	0.01^c^	
			Comprehensibility	3.00 (2.00–3.00)	3.00 (2.75–3.00)	0.19		
Q11. Is long‐term use of antihistamines safe for infants, young children, and pregnant women? If ineffective, can treatment be escalated to omalizumab as in conventional therapy?	Response summary			No definitive answer was given, only a list of the safety and side effects of antihistamines, and the indications for omalizumab, totaling 828 words.	The safety of short‐term and long‐term use of antihistamines in infants, young children, and pregnant women, respectively, was discussed, but no definitive answer was given, totaling 345 words.			
	Evaluation	Dermatologist	Simplicity	2.00 (2.00–3.00)	3.00 (2.00–3.00)	0.07	0.06	0.006**
			Accuracy	3.00 (2.00–3.00)	3.00 (2.00–3.00)	0.35		
			Completeness	3.00 (2.00–3.00)	3.00 (3.00–3.00)	0.07		
			Professionalism	3.00 (2.00–3.00)	3.00 (3.00–3.00)	0.15		
			Cutting‐edge	3.00 (2.00–3.00)	2.00 (2.00–3.00)	0.13		
			Feasibility	3.00 (2.00–3.00)	3.00 (2.00–3.00)	0.66		
		Non‐dermatologist	Simplicity	3.00 (2.00–3.00)	3.00 (2.00–3.00)	0.12	0.04^c^	
			Comprehensibility	3.00 (2.00–3.00)	3.00 (2.75–3.00)	0.19		
Q12. What alternative treatments are available for chronic spontaneous urticaria if omalizumab proves ineffective?	Response summary			The answer contained an obvious contradiction to the established clinical guidelines: the use of antihistamines. Other treatments included immunosuppressants, biologics, herbs, topical, and phototherapy, with no mention of novel monoclonal antibodies such as JAK, BTK, and anti‐ckit, in 927 words.	Other treatments included biologics, immunosuppressants, anti‐inflammatory drugs, other antihistamines, phototherapy, lifestyle, psychological support, but no mention of the novel monoclonal antibodies such as JAK, BTK, anti‐ckit, etc., in 651 words.			
	Evaluation	Dermatologist	Simplicity	2.00 (2.00–3.00)	3.00 (2.00–3.00)	0.04^a^	0.05	0.005**
			Accuracy	3.00 (2.00–3.00)	3.00 (2.00–3.00)	0.27		
			Completeness	3.00 (2.00–3.00)	3.00 (2.00–3.00)	0.65		
			Professionalism	3.00 (2.00–3.00)	3.00 (3.00–3.00)	0.19		
			Cutting‐edge	3.00 (2.00–3.00)	2.00 (2.00–3.00)	0.95		
			Feasibility	3.00 (2.00–3.00)	3.00 (2.00–3.00)	0.92		
		Non‐dermatologist	Simplicity	3.00 (1.00–3.00)	3.00 (2.00–3.00)	0.16	0.03^c^	
			Comprehensibility	3.00 (1.75–3.00)	3.00 (2.00–3.00)	0.10		

*Note:* Values are median (interquartile range). * represents the statistical difference among all evaluators' assessments of the same question. ^a^, ^b^, ^c^, *p < 0.05; ^aa^, ^bb^, ^cc^, **p < 0.01; ^aaa^, ^bbb^, ^ccc^, ***p < 0.001.

^a^ represents the statistical difference among evaluations of the same question under the same assessment criterion.

^b^ represents the statistical difference among all dermatologists' evaluations of a single question.

^c^ represents the statistical difference among all non‐dermatologists' evaluations of a single question.

Importantly, according to the most updated guideline, ChatGPT‐4o made obvious mistakes in three questions: question 6, question 9 and question 12, while DeepSeek‐R1 did not make obvious mistakes.

DeepSeek‐R1's mean scores for each question outperformed those of ChatGPT‐4o, with statistically significant differences observed in all 12 questions (all *p* value < 0.05). For the evaluation metrics of each sub‐item, only 2 sub items were better for ChatGPT than DeepSeek, and both were degree of cutting‐edge (Q11, Q12). 20 of DeepSeek's median (IQR) scores were 3.00 (3.00–3.00) in all 96 reviews, while ChatGPT receive none such score.

### 3.4 Dermatologists' Evaluation

For each evaluation indicator, the responses to the 12 questions by all participated dermatologists were combined for comparison.

**FIGURE 2:**
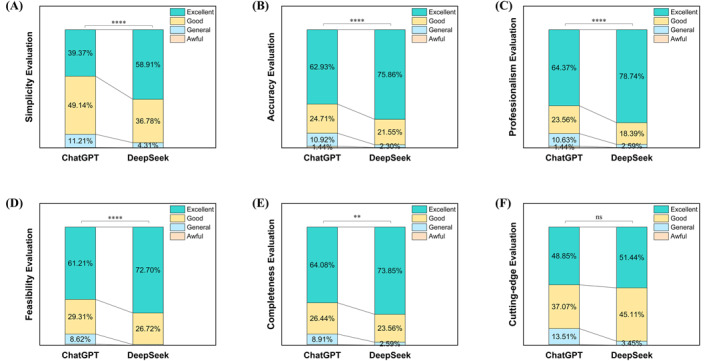
Dermatologists' ratings of responses generated by DeepSeek‐R1 and ChatGPT‐4o. Subgroup analysis of dermatologist scores across individual domains: (A) simplicity, (B) accuracy, (C) professionalism, (D) clinical feasibility, (E) completeness, and (F) integration of cutting‐edge knowledge. ns *p* > 0.05, *p < 0.05, **p < 0.01.***p < 0.001. ****p < 0.0001.

In the simplicity evaluation (Figure 2A), the highest proportion of DeepSeek was Excellent ratings (58.91%), while ChatGPT's highest rating was Good (49.14%). Only ChatGPT got 1 Awful, while it also had 11.21% General ratings, compared to only 4.31% General for DeepSeek. A significant difference was observed between the two models in terms of simplicity (*p* < 0.001).

For Accuracy (Figure 2B), both models received the highest proportion of Excellent ratings, with 62.93% for ChatGPT and 75.86% for DeepSeek. For other grades, ChatGPT received 24.71% Good, 10.92% Normal, and 1.44% Awful ratings. In contrast, DeepSeek had fewer ratings in low grades, with 21.55% Good, 2.30% Normal, and 0.29% Awful. As well, significantly better performance was observed with DeepSeek (*p* < 0.001).

Similar to indicators mentioned before, significant difference in professionalism was observed (*p* < 0.001). Both models received the highest proportion of Excellent ratings among all evaluation metrics, with 64.37% for ChatGPT and 78.74% for DeepSeek (Figure 2C). ChatGPT received 23.56% Good, 10.63% Normal, and 1.44% Awful ratings, while DeepSeek received 18.39% Good, 2.59% Normal, and 0.29% Awful.

In terms of feasibility in clinic practice (Figure 2D), the two received similar percentages of Good ratings (29.31% vs. 26.72%). The distribution of other levels was different, account for the significant difference in distribution between ChatGPT and DeepSeek (*p* < 0.001). Excellent was the most frequently rated in both models, with 61.21% for ChatGPT and 72.70% for DeepSeek. ChatGPT received 8.62% Normal and 0.86% Awful ratings, while DeepSeek had only 0.58% Normal ratings.

Although DeepSeek‐R1 offered greater simplicity, it also provided higher completeness (Figure 2E). The proportion of level 2 (“good”) ratings for both was similar, with the main difference lying in the evaluations of 3 and 1: ChatGPT received 64.08% Excellent and 8.91% Normal, while DeepSeek received 73.85% Excellent and 2.59% Normal. There as a statistically significant difference in the completeness of responses between the two models (*p* = 0.001).

There were no significant differences between the two models in terms of degree of cutting‐edge knowledge (Figure 2F), both models received fewer Excellent ratings compared to other metrics, with 48.85% for ChatGPT and 51.44% for DeepSeek. ChatGPT also received 37.07% Good, 13.51% Normal, and 0.57% Awful ratings, while DeepSeek received 45.11% Good and 3.45% Normal ratings. No significant differences were observed in degree of cutting‐edge knowledge (*p* = 0.06), indicating comparable performance between the two models in these dimensions.

### 3.5 Non‐Dermatologists’ Evaluation

The responses generated by the two AI models were evaluated by non‐dermatologists for simplicity and comprehensibility, with answers from all non‐dermatologist participants across all questions included in the data analysis.

**FIGURE 3:**
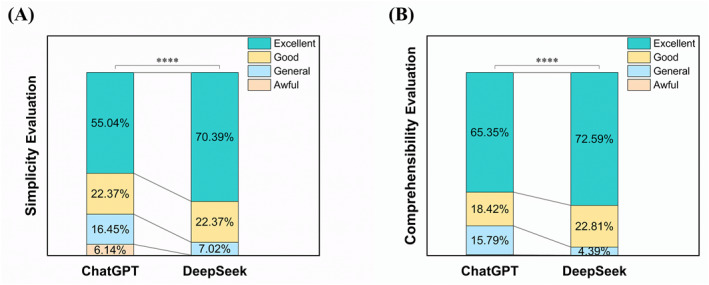
Non‐dermatologists’ ratings of responses generated by DeepSeek‐R1 and ChatGPT‐4o. Evaluation of (A) simplicity and (B) comprehensibility by non‐dermatologist participants. ****p < 0.0001.

In terms of simplicity (Figure 3A), ChatGPT received more Awful and Normal ratings than DeepSeek: 6.14% were Awful, 16.45% were Normal, 22.37% were Good, and 55.04% were Excellent. In contrast, DeepSeek received 70.39% Excellent, 22.37% Good, 7.02% Normal, and only 0.22% Awful rating, with a highly significant difference observed between the two models (*p* < 0.001).

For comprehensibility (Figure 3B), DeepSeek also demonstrated higher scores, with 72.59% Excellent, 22.81% Good, 4.39% Normal, and 0.22% Awful rating, while ChatGPT received 65.35% Excellent, 18.42% Good, 15.79% Normal, and 0.44% Awful ratings, showing a significant difference between the two models (*p* < 0.001).

## 4 Discussion

The evaluation of DeepSeek‐R1 was overall superior to ChatGPT‐4o, with the largest difference between the two being in non‐dermatologists’ ratings of simplicity (*p* < 0.001), and the smallest being the degree of cutting edge (*p* = 0.06). Of interest, the IQR of DeepSeek is less than or equal to the IQR of ChatGPT in each question metric, suggesting better stability of DeepSeek's output. This finding statistically validates DeepSeek's dual advantages in terms of generation quality and reliability.

In the evaluation by professional dermatologists, DeepSeek was superior across the board, with significantly higher scores than ChatGPT for simplicity, accuracy, professionalism, completeness and clinical feasibility (all *p*‐values < 0.05). Especially in terms of simplicity (*p* < 0.001) and professionalism (*p* < 0.001), DeepSeek demonstrated stronger integration of medical knowledge by providing more accurate and detailed disease categorization, pathogenesis analysis, and specific clinical tools. Although the two LLMs did not show significant differences in degree of cutting‐edge, DeepSeek had better control of low‐scoring evaluation (General or Awful) (DeepSeek vs. ChatGPT: 3.45% vs. 14.08%).

It is worth noting that ChatGPT showed obvious “contradictions to established clinical guidelines” in the answers to three questions (related to classification, treatment, and diagnosis), while DeepSeek did not show such problems, suggesting that DeepSeek may be more rigorous in the construction and validation mechanism of medical knowledge base.

In the evaluation by non‐dermatologists, DeepSeek's higher evaluation of simplicity and comprehensibility indicated the general applicability of its user‐friendly design to the general public. Especially in the evaluation of simplicity, the difference bifurcated more significantly (ChatGPT low‐score evaluation rate 22.58% vs. DeepSeek 7.24%), which may suggest that the LLMs need to be adapted to the representation strategies of different knowledge backgrounds.

The two LLMs also showed different characteristics for different types of questions. In the diagnostic decision category of questions (e.g., Q9 treatment approach), DeepSeek‐R1 significantly improved the clinical feasibility score through structured response (adding scale/subpopulation discussion). In the basic mechanism category of questions (e.g., Q3 Pathogenesis): ChatGPT‐4o countered in the simplicity score (*p* = 0.12), suggesting that there is room for optimizing the simplified expression of complex medical concepts. As for the questions in the special population category (e.g., Q11 and Q12 medication selection), both models showed a trough in the frontier score, exposing a lag in tracking the latest guidelines.

As seen from the question specificity analysis, DeepSeek excelled in handling complex clinical decision‐making problems, such as obtaining the highest completeness score by stratifying the discussion of population‐specific risks and alternative treatment options in the answer to “Long‐term use of antihistamines in infants, children, and pregnant women” (Q11). This suggests that the structured output pattern of the models within the framework of evidence‐based medicine is more in line with clinical needs. On the contrary, ChatGPT‐4o's got the lowest score for Q9 (treatment options), exposed its shortcomings in multi‐option prioritization with evidence level annotation. When developing medical‐specific AIs in the future, the ability to parse clinical guidelines should be strengthened, and a dynamically updated warning mechanism for adverse reactions should be established.

Various investigations have been conducted on the feasibility of the use of large language models in clinical settings.

The study in 2023 by Goodman et al. [13] assessed the professionalism and accuracy of ChatGPT‐3.5 and ChatGPT‐4 in answering different types and difficulties of medical questions. The results showed that ChatGPT made several striking and surprising “contradictions to established clinical guidelines” and was less accurate for more difficult questions, demonstrating the limitations of LLMs in dealing with complex medical questions, and thus the researchers suggested that the use of ChatGPT 3.5/4 may not be desirable for medical knowledge dissemination yet.

In the same year, Young et al.'s study [14] evaluated the performance of ChatGPT‐4 in answering melanoma‐related questions. The results of the study suggested that the average readability of ChatGPT‐4 responses corresponded to college‐level comprehension and was too advanced and difficult to understand for the general public. This is consistent with the comprehensibility results evaluated by non‐dermatologists in this study.

In 2024, a study by Naweed Shifai et al. [15] examined the ability of ChatGPT Vison to recognize and diagnose dermoscopic melanoma images, and demonstrated that ChatGPT Vision was significantly below the level of performance demonstrated by current market‐recognized AI algorithms in diagnosing melanoma based on dermoscopic images. It had considerable risk of missing melanomas and incorrectly classifying lesions as malignant. These findings are consistent with the results of the present study, suggesting that ChatGPT may make some wrong responses and poor accuracy.

These studies have shown that ChatGPT 4/4o has some limitations, especially in terms of accuracy, correctness, and ease of understanding of responses, which are consistent with the results of this study. The present study shows that DeepSeek‐R1 has improved in all these aspects, and the emergence of DeepSeek‐R1 nowadays is undoubtedly a great improvement on the previous lack of accuracy and correctness of ChatGPT‐4o.

Peng et al. [16] suggested that DeepSeek used large‐scale reinforcement learning, reward modeling, and distillation to improve inference performance and that its “critical thinking” functionality can be made more accessible and understandable through incremental logical reasoning, which may be one of the reasons why DeepSeek‐R1 performs better than ChatGPT‐4o.

Semeraro et al. [17] examined the accuracy, clarity, explanation of technical terms, and references to guideline updates in CRP presentation and interpretation by different AI tools. The results mentioned that ChatGPT‐4o and DeepSeek‐R1 had good accuracy, while ChatGPT was more concise and easier to understand compared to DeepSeek. It is somewhat different from the results of this study and may be related to the direction of specialization, input instructions, and number of questions.

The auxiliary function of DeepSeek may be better in clinical diagnosis and treatment due to DeepSeek's high accuracy, zero rate of answers contradictions to established clinical guidelines, and stronger clinical practicability, which can be an important tool for rapid retrieval of medical information in a fast‐paced clinical environment in order to improve the efficiency of diagnosis and treatment. When it comes to patients' understanding of cognitive diseases, DeepSeek is more practical because it is highly concise and easy to understand. As for the retrieval of scientific information, ChatGPT‐4o may show some advantages, but there is a possibility of false information and misinformation, which needs to be carefully screened. For cutting‐edge scientific information, both processing and answering of the LLMs are lacking, which may be due to the fact that they are updating the information through web learning, and untimely updating may cause delays in the content of the models. However, it is still unknown whether these deficiencies of LLMs can be improved by continuous updating and improvement of the algorithms as well as by repeated user feedback through reinforcement learning.

It is important to note that this study was conducted in Chinese and targeted native Chinese speakers, whereas GPT‐4o was primarily trained on English‐language data. This language discrepancy may have introduced bias in the model's responses. In contrast, DeepSeek‐R1 was trained on a larger proportion of Chinese‐language data, which may partially explain its comparatively better performance in this setting. These findings underscore the importance of comparing multiple AI tools in multilingual contexts. Moreover, future work should explore the use of fine‐tuning and retrieval‐augmented generation (RAG) to optimize AI‐generated responses for specific domains and languages.

Large language models may become an important tool in the future to level out unequal information (information gap) between doctors and patients. On the one hand, it may facilitate communication between doctors and patients in the clinic. On the other hand, it may demand a higher level of professional competence for doctors. As Carl Preiksaitis put it [18], “Generative AI has the potential to revolutionize many aspects of medicine, but relying on this approach alone may ultimately not ease the burden on physicians and other clinicians, and may even make things worse.”

A limitation of the current study is that responses were obtained via web interfaces rather than application programming interface, without controlling model parameters or averaging multiple runs, which may affect reproducibility due to the stochastic nature and continuous updates of the models. No custom instructions or prompt wording were provided when posing questions, potentially limiting reproducibility. Reproducibility might be improved by applying specific constraints and averaging multiple runs in future research. The assessment of “cutting‐edge knowledge” was based on alignment with the 2022 EAACI/GA^2^LEN guidelines, but this criterion may not comprehensively capture all recent advances. The “Simplicity” metric assessed in this study may correlate with response length. Although the scoring guidelines require distinguishing between effective content simplification and mere textual refinement, we cannot entirely rule out the possibility of evaluators being subconsciously influenced by length, which may constitute a potential confounding factor. The current grading criteria fail to distinguish between major and minor factual errors, resulting in certain limitations. Future research should establish a more detailed error classification system and incorporate multiple independent reviewers to enhance the transparency and reliability of the evaluation process. Furthermore, differences in model performance may be influenced by varying knowledge cut‐off dates, which limit direct comparability. Model comparisons in this study are based on API availability as of February 2025. Future research should include updated models (such as GPT‐5 and DeepSeek‐V3) for more comprehensive evaluation. Additionally, this study did not assess the ability of LLMs to integrate graphic and text outputs, such as recognizing or diagnosing skin disease images. The number of evaluated questions was limited, and binary question types were underrepresented. Furthermore, the participant demographics, including age, gender, and geographic distribution, were skewed, potentially impacting the generalizability of the findings. Moreover, a potential limitation is the risk of confirmation bias, as the same team created both the questions and answers; future studies should use a double‐blind design with independent teams will minimize this bias.

Overall, this comparative study demonstrates that DeepSeek‐R1 outperforms ChatGPT‐4o in addressing urticaria‐related queries across both clinical and non‐specialist contexts. For dermatologists, DeepSeek‐R1 exhibited significantly superior performance in simplicity, accuracy, professionalism, completeness and clinical feasibility, with no answers contradictions to established clinical guidelines observed in diagnostic or treatment‐related responses—a critical advantage over ChatGPT‐4o, which made notable answers contradictions to established clinical guidelines in three clinical questions. However, limitations persist. Both models lagged in tracking the latest guideline updates, underscoring the need for dynamic, real‐time knowledge refinement mechanisms. Despite these challenges, DeepSeek‐R1 emerges as a promising tool for enhancing clinical decision‐making and patient education, offering precise, actionable, and accessible information.

## Author Contributions

**Mengyao Yang:** writing – original draft, software. **Jingchen Liang:** investigation. **Luyue Zhang:** validation. **Hongshan Liu:** validation. **Ying Chen:** data curation. **Yawen Wang:** formal analysis. **Chunzhi Qi:** investigation. **Yuxin Ma:** investigation. **Ziyun Gao:** investigation. **Xinyue Zhang:** methodology. **Xinwu Niu:** methodology. **Xiaopeng Wang:** methodology. **Jianwen Ren:** methodology. **Jingyi Yuan:** methodology. **Weihui Zeng:** supervision, funding acquisition. **Zhao Wang:** funding acquisition, supervision, writing – review and editing, investigation.

## Funding

This study was supported by the National Natural Science Foundation of China (NSFC) (82201966) and Natural Science Basic Research Program of Shaanxi (2023JC‐QN‐0924) to Zhao Wang, Xi'an Science and Technology Plan Project (2023JH‐YXYB‐0009), Xi'an Jiaotong University Medical Development Fund (XJYG2025‐SFJJ039) to Weihui Zeng. Natural Science Basic Research Program of Shaanxi (2023‐JC‐QN‐0955) to Xinyue Zhang.

## Conflicts of Interest

The authors declare no conflicts of interest.

## Supporting information


Supporting Information S1

Supporting Information S2

**Table S1:** Scoring results of all the questions for assessing AI models capability. Detailed display of the scores for each question when developing the questionnaire. A panel of 30 dermatologists evaluated the relevance of each question from clinical practice, clarity of expression, and patient attention. The evaluation of each question is divided into 5 levels (5 = strongly agree, 4 = agree, 3 = uncertain, 2 = disagree, 1 = strongly disagree). The “total score” is the sum of all respondents' ratings on the question, and the “average score” is the arithmetic mean of the total scores.

## Data Availability

The data that support the findings of this study are available from the corresponding author upon reasonable request.

## References

[1] T. Zuberbier , A. H. Abdul Latiff , M. Abuzakouk , et al., “The International EAACI/GA(2)LEN/EuroGuiDerm/APAAACI Guideline for the Definition, Classification, Diagnosis, and Management of Urticaria,” Allergy 77, no. 3 (2022): 734–766, 10.1111/all.15090. 34536239

[2] J. Fricke , G. Avila , T. Keller , et al., “Prevalence of Chronic Urticaria in Children and Adults Across the Globe: Systematic Review With Meta‐Analysis,” Allergy 75, no. 2 (2020): 423–432, 10.1111/all.14037. 31494963

[3] Y. Rosman , A. Y. Hershko , K. Meir‐Shafrir , et al., “Characterization of Chronic Urticaria and Associated Conditions in a Large Population of Adolescents,” Journal of the American Academy of Dermatology 81, no. 1 (2019): 129–135, 10.1016/j.jaad.2019.02.034. 30797847

[4] M. Maurer , P. Kolkhir , M. P. Pereira , et al., “Disease Modification in Chronic Spontaneous Urticaria,” Allergy 79, no. 9 (2024): 2396–2413, 10.1111/all.16243. 39044706

[5] R. Asero , M. Ferrer , E. Kocaturk , and M. Maurer , “Chronic Spontaneous Urticaria: The Role and Relevance of Autoreactivity, Autoimmunity, and Autoallergy,” Journal of Allergy and Clinical Immunology: In Practice 11, no. 8 (2023): 2302–2308, 10.1016/j.jaip.2023.02.022. 36868473

[6] T. Zuberbier , L. F. Ensina , A. Gimenez‐Arnau , et al., “Chronic Urticaria: Unmet Needs, Emerging Drugs, and New Perspectives on Personalised Treatment,” Lancet 404, no. 10450 (2024): 393–404, 10.1016/s0140-6736(24)00852-3. 39004090

[7] A. Kaplan , M. Lebwohl , A. M. Gimenez‐Arnau , M. Hide , A. W. Armstrong , and M. Maurer , “Chronic Spontaneous Urticaria: Focus on Pathophysiology to Unlock Treatment Advances,” Allergy 78, no. 2 (2023): 389–401, 10.1111/all.15603. 36448493

[8] N. H. Shah , D. Entwistle , and M. A. Pfeffer , “Creation and Adoption of Large Language Models in Medicine,” JAMA 330, no. 9 (2023): 866–869, 10.1001/jama.2023.14217. 37548965

[9] M. H. Temsah , A. Jamal , K. Alhasan , A. A. Temsah , and K. H. Malki , “OpenAI o1‐Preview Vs. ChatGPT in Healthcare: A New Frontier in Medical AI Reasoning,” Cureus 16, no. 10 (2024): e70640, 10.7759/cureus.70640. 39359332 PMC11444422

[10] X. Bi , D. Chen , G. Chen , et al., “DeepSeek LLM: Scaling Open‐Source Language Models With Longtermism,” *arXiv* (2024).

[11] Q. N. Hong , P. Pluye , S. Fabregues , et al., “Improving the Content Validity of the Mixed Methods Appraisal Tool: A Modified e‐Delphi Study,” Journal of Clinical Epidemiology 111 (2019): 49–59e1, 10.1016/j.jclinepi.2019.03.008. 30905698

[12] G. Norman , “Likert Scales, Levels of Measurement and the ‘Laws’ of Statistics,” Advances in Health Sciences Education: Theory and Practice 15, no. 5 (2010): 625–632, 10.1007/s10459-010-9222-y. 20146096

[13] R. S. Goodman , J. R. Patrinely , C. A. Stone Jr. , et al., “Accuracy and Reliability of Chatbot Responses to Physician Questions,” JAMA Network Open 6, no. 10 (2023): e2336483, 10.1001/jamanetworkopen.2023.36483. 37782499 PMC10546234

[14] J. N. Young , O. H. Ross , D. Poplausky , et al., “The Utility of Chatgpt in Generating Patient‐Facing and Clinical Responses for Melanoma,” Journal of the American Academy of Dermatology 89, no. 3 (2023): 602–604, 10.1016/j.jaad.2023.05.024. 37207953

[15] N. Shifai , R. Van Doorn , J. Malvehy , and T. E. Sangers , “Can ChatGPT Vision Diagnose Melanoma? an Exploratory Diagnostic Accuracy Study,” Journal of the American Academy of Dermatology 90, no. 5 (2024): 1057–1059, 10.1016/j.jaad.2023.12.062. 38244612

[16] Y. Peng , B. A. Malin , J. F. Rousseau , et al., “From GPT to DeepSeek: Significant Gaps Remain in Realizing AI in Healthcare,” Journal of Biomedical Informatics 163 (2025): 104791, 10.1016/j.jbi.2025.104791. 39938624 PMC12188495

[17] F. Semeraro , M. Cascella , J. Montomoli , V. Bellini , and E. G. Bignami , “Comparative Analysis of AI Tools for Disseminating CPR Guidelines: Implications for Cardiac Arrest Education,” Resuscitation 208 (2025): 110528, 10.1016/j.resuscitation.2025.110528. 39909198

[18] C. Preiksaitis , C. A. Sinsky , and C. Rose , “ChatGPT Is Not the Solution to Physicians' Documentation Burden,” Nature Medicine 29, no. 6 (2023): 1296–1297, 10.1038/s41591-023-02341-4. 37169865

