# Supplementary material for: Evaluating Generative AI Large Language Models for Urticaria Management: A Comparative Analysis of DeepSeek‐R1 and ChatGPT‐4o
Source: Clin Transl Allergy. 2025 Nov 27;15(11):e70113. doi: 10.1002/clt2.70113 (PMC12658338; doi:10.1002/clt2.70113)
Supplement: Supplementary file 1 — Supporting Information S1 [file CLT2-15-e70113-s001.docx]

**Responses From ChatGPT-4.0**

**Q1. What is urticaria?**

Urticaria, also known as rubella or hives, is a common skin allergic reaction that manifests itself as sudden red, itchy, windy bumps on the skin (similar to lumps from mosquito bites). These wind bumps are usually caused by skin sensitization to certain irritants and are often accompanied by an itching sensation or burning sensation.

The main symptoms of hives include:

Red or whitish-skinned bumps on the skin, which can vary in size and shape and usually disappear within a few hours.

An itching sensation, sometimes accompanied by a slight burning sensation or pain.

Localized swelling that may occur on the lips, eyelids, hands and feet.

Common triggers of hives include:

Food allergies: such as seafood, nuts, and dairy products.

Drug allergies: certain medications may cause an allergic reaction.

Temperature changes: hot and cold stimuli, sweat, etc.

Insect bites: such as mosquitoes, bees, etc.

Stress or mood swings: some people may break out in hives when they are emotionally stressed or under pressure.

Infections: such as an immune response caused by a viral or bacterial infection.

Physical stimuli: such as friction, sun exposure, cold wind, etc.

Classification of hives:

Acute hives: symptoms usually last less than 6 weeks and are often caused by some external factor.

Chronic urticaria: Symptoms last more than 6 weeks, sometimes the cause is difficult to pinpoint and may be related to an abnormality in the immune system.

Treatment:

Antihistamines: commonly used to relieve symptoms and reduce the appearance of itching and windburn.

Allergen avoidance: Finding and avoiding substances or environmental factors that may cause allergies.

Topical treatments: such as the use of creams containing corticosteroids to help reduce symptoms.

Severe cases: For severe symptoms triggered by an acute allergic reaction, epinephrine injections or other first aid measures may be needed.

If symptoms persist or are very severe, it is best to see a doctor for professional diagnosis and treatment.

**Q2. How is urticaria classified?**

Hives can be categorized into two main groups based on the duration and cause of symptoms: acute hives and chronic hives. In addition, urticaria can be further subdivided according to triggers or specific symptoms.

1. Acute Urticaria (Urticaria Acute)

Duration: Usually less than 6 weeks, in most cases subsides on its own within a few hours to a few days.

Symptoms: The appearance of sudden, red, swollen masses of wind, often accompanied by intense itching. The windmills change and move constantly, and may not leave any traces when they subside.

Common Causes: Allergic reactions are the main cause of acute urticaria, which may be caused by food, medication, insect bites, environmental factors (e.g., hot and cold stimuli), and infection.

2. Chronic Urticaria

Duration: lasts for more than 6 weeks, and sometimes symptoms may last for months or even years.

Symptoms: Symptoms are similar to those of acute urticaria, but with a higher frequency of the eruption, there may be no obvious triggers, and symptoms may continue to recur.

Cause: The cause of chronic urticaria is not easy to pinpoint and may be related to factors such as abnormalities in the immune system, chronic illness, and mental stress. In some cases, chronic urticaria is associated with the body's autoimmune response.

3. Special types of urticaria

In addition to acute and chronic urticaria, there are special types of urticaria which are categorized according to specific triggers and symptoms:

Physical urticaria:

Cold urticaria: symptoms of urticaria appear after stimulation by cold air, cold water, etc..

Heat urticaria: caused by heat, exercise, etc.

Compression urticaria: caused by localized pressure (e.g., tight clothing, prolonged sitting).

Solar urticaria: Symptoms can be triggered by exposure to sunlight and are common in sunny weather.

Angioedema (angioedema): this is a deeper skin reaction usually accompanied by more severe swelling, especially in areas such as the eyelids, lips, and throat, which may lead to breathing difficulties. Although angioedema often occurs with urticaria, it is a different condition in its own right.

Drug urticaria: an allergic reaction caused by medications. Common medications include antibiotics, NSAIDs (non-steroidal anti-inflammatory drugs), and aspirin.

Food urticaria: allergic reactions triggered by certain foods (e.g. nuts, seafood, dairy products, etc.).

Autoimmune hives: in some cases, an abnormal immune system may cause the body to develop an immune response to its own tissues, resulting in the appearance of chronic hives.

4. Hives accompanied by other symptoms

Some people with hives may experience symptoms from other systems, such as joint pain, fever, nausea, etc. These conditions usually require medical tests to screen for other diseases, especially autoimmune diseases.

To summarize:

Urticaria can be divided into two main categories, acute and chronic, and specific treatments and management will vary depending on the type of urticaria. Acute hives are usually caused by external allergens and treatment is mostly based on antihistamines, while chronic hives may require more in-depth investigations to look for underlying causes and long-term management measures.

**Q3. What causes urticaria?**

Hives (also called rubella) is a skin condition that usually manifests itself as red, itchy, windy bumps or patches on the skin that may be accompanied by swelling. This condition can occur for a variety of reasons, including the following:

Allergic reactions:

Hives are usually caused by an allergic reaction of the immune system to certain external stimuli. Common allergens include:

Foods: such as seafood, nuts, eggs, and milk.

Medications: e.g. certain antibiotics, non-steroidal anti-inflammatory drugs (NSAIDs), etc.

Insect bites: e.g. mosquito, bee bites, etc.

Environmental allergens: such as pollen, dust mites, animal dander, etc.

Physical Factors:

Hives are sometimes associated with physical stimuli, for example:

Hot or cold stimuli: e.g. cold air, warm water, sweating after strenuous exercise may trigger hives.

Pressure or friction: sitting or standing for long periods of time or wearing clothes that are too tight can also trigger it.

Sun exposure: Some people may be sensitive to ultraviolet rays and tend to develop hives after exposure to sunlight.

Infections:

Some viral, bacterial or fungal infections can also trigger hives. For example, flu, colds, streptococcal infections, etc.

Endocrine disorders:

The appearance of hives is also related to the body's hormonal changes, especially in cases of stress, mood swings, menstrual cycle, etc., the immune system will undergo some changes that may trigger hives.

Autoimmune diseases:

Some autoimmune diseases, such as lupus erythematosus and rheumatoid arthritis, may also be related to the occurrence of urticaria.

Genetic factors:

Individuals may also be more susceptible to hives if there is a family history of allergic diseases such as allergic rhinitis and asthma.

Overall, the causes of hives are complex and may be a result of a single factor or a combination of factors. If you experience symptoms of hives, it is best to consult a dermatologist for diagnosis and treatment so that you can understand the exact cause of the condition and take appropriate treatment measures.

**Q4. How is urticaria diagnosed?**

Diagnosis of urticaria is usually based on a detailed history, clinical presentation, and the necessary ancillary tests. The following are the main steps in the diagnosis of urticaria:

1. History inquiry

Symptomatic manifestations: Your doctor will ask you in detail about the symptoms of your skin lesions, such as the time of appearance, shape and fading of the windmill. Urticaria is typically characterized by a red or pinkish-red rash with well-defined edges, and will subside on its own within a few hours.

Triggers: Ask if there are any known allergens or triggers, such as food, medications, environmental factors (e.g., pollen, dust, etc.), insect bites, stress, and cold.

Accompanying symptoms: e.g. fever, joint pain, dyspnea, etc. Systemic symptoms may suggest other types of allergic reactions or diseases (e.g. systemic lupus erythematosus, purpura, etc.).

2. Clinical signs

Physical examination: The doctor will observe the pattern of skin eruptions. Usually, urticaria eruptions are raised, red, swollen plaques that are usually itchy when touched.

Fading of the skin tags: The tags of urticaria disappear within a short period of time (usually within 1-24 hours) and do not leave visible skin damage, which helps to differentiate them from other skin diseases (e.g. eczema, skin infections, etc.).

3. Allergen testing

Skin prick test: If a specific allergen (e.g. food, pollen, dust mite, etc.) is suspected to be the cause of the allergy, your doctor may recommend allergen testing. By pricking extracts of different allergens on a small area of the skin, an allergic reaction is observed.

Blood tests: e.g. total IgE level, specific IgE antibody test, these can help to determine whether there is an allergic reaction, especially in the case of allergic urticaria.

4. Other laboratory tests

If your doctor suspects that urticaria may be associated with certain systemic diseases or infections, he or she may recommend other tests, such as:

Blood tests: to check for an elevated white blood cell count to rule out bacterial infections.

Liver and kidney function tests: to check for abnormal liver and kidney function, especially if certain medications or autoimmune diseases are suspected.

Antinuclear antibody (ANA) test: This may be performed if hives are suspected to be associated with an immune disorder such as systemic lupus erythematosus.

5. Ruling out other diseases

The diagnosis of urticaria is usually arrived at after ruling out other similar diseases. The doctor will rule out other possible skin diseases such as contact dermatitis, eczema, drug rashes, skin infections, etc. based on the clinical presentation.

Diagnosis of chronic urticaria: Chronic urticaria is diagnosed if urticaria symptoms persist for more than 6 weeks and recur. Chronic urticaria may be associated with autoimmune factors.

6. Trigger tests (e.g., physical hives)

For certain types of hives (e.g., pressure hives, cold hives, etc.), your doctor may perform specific trigger tests:

Cold test: to see if hives are triggered by exposing the skin to cold temperatures.

Pressure test: to see if hives are produced by applying localized pressure (e.g., by pressing on the skin).

Sunlight test: check for a rash by exposing the skin to ultraviolet light.

7. Self-observation and documentation

During the diagnostic process, the doctor may advise the patient to document possible triggers, such as foods, medications, environmental changes, etc., to help identify specific allergens or triggers.

8. Differential diagnosis

As many skin diseases have similar symptoms, doctors need to differentiate them from other similar diseases (e.g. contact dermatitis, eczema, drug allergy, and purpura, etc.) to ensure an accurate diagnosis.

To summarize:

The diagnosis of urticaria is usually made by a detailed history, clinical presentation, and necessary ancillary tests. Most cases of urticaria are diagnosed by exclusion, targeting possible allergens and triggers to help determine the cause. During treatment, the doctor may also advise the patient to avoid known allergens and triggers.

**Q5. Are laboratory tests necessary for diagnosing acute urticaria?**

Acute hives (also known as acute urticarial reaction or hives flare-ups) usually appear as red, itchy windy bumps on the skin and can be caused by a variety of factors, including allergies, infections, drug reactions, and food. The need for laboratory tests depends on the severity of the symptoms, the suspected cause, and the judgment of the doctor.

1. Common Laboratory Tests

Laboratory tests are not usually routinely necessary for acute urticaria, but in some cases, your doctor may recommend the following tests:

Routine blood tests: These can check for signs of an allergic reaction or infection. For example, if an allergic reaction due to infection is suspected, the white blood cell count may be elevated.

Allergen screening: If hives are suspected to be caused by an allergy, your doctor may recommend allergen testing, such as a skin prick test or specific IgE antibody test (e.g., food allergy, drug allergy, etc.).

Liver, kidney, and thyroid function tests: In some cases, especially if the hives are accompanied by other symptoms (e.g., weight changes, swelling, etc.), your doctor may recommend tests of liver and kidney function or thyroid function to rule out other underlying conditions.

Immunologic tests: If an allergic reaction due to autoimmunity is suspected, some immunologic tests may be done.

2. When laboratory tests are needed

If the symptoms of hives last for a long time (more than 6 weeks), further tests may be needed to rule out chronic hives or other underlying health problems.

If hives are suspected to be related to a systemic disease (such as an endocrine or immune disorder), additional laboratory tests may be needed.

If symptoms are accompanied by a severe allergic reaction, such as shortness of breath and swelling of the throat, urgent medical attention and related tests may be required.

3. Routine management

Most cases of acute urticaria can be diagnosed by assessment of clinical symptoms and laboratory tests are often not required. Treatment is usually based on antihistamines to help relieve itching and redness. If the allergen is not clear, the physician may make a preliminary judgment based on the history and clinical presentation.

To summarize:

Acute urticaria usually does not require routine laboratory tests, but depending on the complexity of the condition and the suspected cause, your doctor may recommend some laboratory tests to help confirm the cause or rule out other underlying conditions.

**Q6. What are the causes of chronic spontaneous urticaria, and how is it classified?**

Chronic Spontaneous Urticaria (CSU) is a common skin condition that presents with recurring windburns (i.e., red or white bumps of skin) and intense itching that lasts for more than 6 weeks. The specific etiology can be complex and involves a variety of factors, here are some possible causes and ways to categorize them:

1. Causes of chronic spontaneous urticaria:

The exact cause of chronic spontaneous urticaria is not entirely clear, but current research suggests that it may be related to the following factors:

Abnormalities of the immune system: Some studies suggest that chronic spontaneous urticaria may be associated with disorders of the immune system. For example, an autoimmune response may be present in some patients, causing the immune system to mistakenly attack its own skin cells.

Role of mast cells: Mast cells are immune cells in the skin and other tissues that play an important role in allergic reactions. Chronic spontaneous urticaria may be due to the abnormal release of histamine and other inflammatory mediators from mast cells, leading to dilation of blood vessels and the appearance of windswept skin.

Genetic factors: Some studies have found that people with a family history of allergies or hives have a higher risk of developing chronic spontaneous urticaria, suggesting that genetic factors may play a role.

Infections and viruses: some viral (e.g., EBV, hepatitis viruses, etc.) or bacterial infections may trigger hives, especially if the immune system is low.

Environmental factors: Hives may be triggered by factors including climate change, excessive UV exposure, and high or low temperatures.

Medications and foods: Some medications (e.g. antibiotics, painkillers, etc.) and certain foods (e.g. seafood, nuts, etc.) may also be triggers for hives.

Stress and Emotional Factors: Mood swings, excessive stress, anxiety and other factors have also been associated with the development and exacerbation of urticaria in some patients.

2. Classification of chronic spontaneous urticaria:

Chronic spontaneous urticaria can usually be categorized into the following two types:

Type I: idiopathic chronic spontaneous urticaria

The cause of the disease in this group of patients is unclear and is usually thought to be the result of abnormalities in the immune system or overactivation of mast cells. Idiopathic chronic spontaneous urticaria accounts for approximately 75% or more of all patients.

Type 2: Secondary chronic spontaneous urticaria

Secondary urticaria is caused by known external factors such as drugs, infections, food allergies, and endocrine abnormalities. In some cases, other underlying conditions (e.g., thyroid disorders, digestive disorders, etc.) may be triggers of urticaria.

3. Symptoms and clinical manifestations of chronic spontaneous urticaria:

Dermatomes: red or pale, well-defined lumps (dermatomes) appearing on the surface of the skin, which may sometimes be slightly depressed in the center. They vary in size and shape and can be constantly changing.

Itching: Usually accompanied by intense itching, which may cause the patient to scratch, further aggravating the skin damage.

Duration: Chronic spontaneous urticaria can be diagnosed if symptoms persist for more than 6 weeks without a clear trigger.

4. Treatment:

Treatment of chronic spontaneous urticaria usually includes medication and avoidance of triggers:

Antihistamines: These are the drugs of choice for urticaria and reduce symptoms by inhibiting histamine release.

Immunomodulatory drugs: such as anti-interleukin-5 (e.g., omalizumab) and immunosuppressants for patients who have failed to respond to conventional treatment.

Topical treatments: For localized itching and skin lesions, topical medications that calm itching, such as hormonal creams, may be used.

Avoidance of triggers: Avoid known triggers such as specific foods, medications or environmental factors.

Treatment of chronic spontaneous urticaria needs to be individualized, and consultation with a dermatologist or allergist is recommended for diagnosis and treatment.

**Q7. How is the severity and activity of urticaria assessed?**

Hives (also known as rubella) is a common skin condition that usually presents as red, itchy, intensely itchy, windy masses of skin, often accompanied by edema. The severity or activity of hives can generally be assessed by the following:

1. Extent and distribution of symptoms

Mild: If urticaria is confined to a small, localized area with relatively few lesions and resolves on its own within a short period of time, it is considered mild.

Moderate: If the urticaria is present in a larger area and may be accompanied by multiple flare-ups, but the duration of the symptoms is short, it can be considered moderate.

Severe: If the urticaria covers a wide area, the lesions are widely distributed and last for a longer period of time, which may involve several parts or the whole body, it means that the urticaria is more serious and may even be accompanied by systemic symptoms.

2. Duration of the rash

Acute urticaria: Symptoms usually resolve within 24 hours, and the rash flares up and subsides in one to two days.

Chronic urticaria: If symptoms persist for more than six weeks and recur periodically, they are chronic and may require more aggressive treatment to control symptoms.

3. Frequency of symptoms

Assessment of activity: Activity refers to the frequency and variability of urticaria symptoms. For example, if symptoms recur frequently and each recurrence is more severe, activity is high. On the contrary, if the symptoms recur occasionally and are mild each time, the activity level is low.

4. Accompanying symptoms

With systemic symptoms: If hives are accompanied by severe systemic reactions such as fever, nausea, dizziness, swelling of the throat, and difficulty in breathing, it may be a life-threatening anaphylactic shock caused by an allergic reaction, and requires prompt medical attention.

No systemic symptoms: only localized skin symptoms, usually a milder type, not accompanied by systemic problems, easier to control.

5. Degree of itching

Mild itching: If the itching sensation is mild and the area is not easily scratched, it may indicate mild symptoms.

Intense itching: If the symptoms are accompanied by intense itching, and scratching is likely to cause skin breakdown or inflammation, this is usually a sign of more severe symptoms and may exacerbate the condition.

6. Other comorbidities

If the patient has other chronic diseases, such as asthma and allergic rhinitis, this may make it more difficult to control the hives and make the symptoms more likely to recur.

7. Drug reactions

Drug control: If symptoms are relieved with antihistamines, steroids, or other anti-allergy drugs, it means that the urticaria can be controlled and the severity is mild.

Drug resistance: If the patient does not respond to conventional drugs and symptoms persist, it may indicate a more severe condition.

Comprehensive Assessment

Based on the above points, you can comprehensively determine the severity and activity of hives. If the symptoms are relatively mild, occasional and easily controlled, they are generally mild. If the symptoms occur frequently and have a large distribution, accompanied by intense itching, they may be moderate or severe, and you need to seek medical help to formulate an appropriate treatment plan.

In case of emergencies such as breathing difficulties and generalized allergic symptoms, seek immediate medical attention.

**Q8. Is urticaria curable?**

Hives (also known as rubella) is a common skin condition that usually manifests itself as a sudden appearance of red, swollen, itchy, windy bumps on the skin that may last anywhere from a few minutes to a few hours. Hives can be caused by many factors, such as food allergies, drug allergies, infections, and stress.

Regarding whether hives can be cured, the situation varies from person to person:

Acute hives: most acute hives will clear up on their own within a few days to a few weeks. Acute hives are often caused by an allergic reaction or some kind of infection, and once the trigger is removed, the symptoms usually go away.

Chronic Hives: Chronic hives have a longer duration, usually lasting 6 weeks or more. Chronic hives may be caused by abnormalities in the immune system, environmental factors, or unspecified causes. In this case, although a complete “cure” may not be possible, most patients can be treated with medications (e.g. antihistamines) to control symptoms and reduce the frequency and severity of flare-ups.

Treatment:

Medications: Commonly used treatments include antihistamines, corticosteroids, and immunosuppressants, which can help alleviate symptoms and reduce itching and swelling.

Avoidance of triggers: Reduce hives flare-ups by avoiding known allergens (e.g., certain foods, medications, climate change, etc.).

Lifestyle modifications: Maintaining a healthy diet, avoiding excessive stress and overheating, and maintaining good habits to help minimize the recurrence of symptoms.

Overall, while acute hives can usually be cured, treatment of chronic hives may require long-term management. Consultation with a dermatologist is recommended to make a diagnosis and develop an appropriate treatment plan.

**Q9. How is urticaria treated?**

Treatment of urticaria depends largely on its type (acute or chronic) and cause. The goal of hives treatment is to relieve symptoms (such as itching and swelling) and to reduce the frequency and severity of flare-ups. Here are some common treatments:

1. Medication

Medication is the most common method of controlling hives and includes the following:

Antihistamines (most commonly used):

Antihistamines are the medications of choice for hives and provide effective relief from itching, swelling and windburn. Common antihistamines include loratadine (Claritin), fexofenadine (Allegra), and cetirizine (Zyrtec).

Second-generation antihistamines: Compared to first-generation antihistamines, second-generation drugs are less likely to cause drowsiness and can therefore be used for extended periods of time.

Steroid medications:

If symptoms are severe and antihistamines are not effective, your doctor may prescribe short-term oral or topical steroids (e.g., prednisone) to reduce the inflammatory response.

Immunomodulatory medications:

For patients with chronic hives or medications that are not effective, your doctor may recommend biologics such as **anti-interleukin-5 (e.g., omalizumab)**. These medications can help regulate the immune system and reduce symptoms.

Antibiotics and antiviral medications:

If hives are caused by a bacterial or viral infection, your doctor may prescribe antibiotics or antiviral medications depending on the type of infection.

2. Avoiding triggers

Some hives are triggered by external factors (e.g. food, medication, climate change, etc.), so avoiding these triggers is one of the keys to treatment:

Food allergy: If you know that you are allergic to certain foods (e.g. seafood, nuts, dairy products, etc.), you should avoid consuming these foods.

Drug allergies: If you have a history of drug allergies, you should avoid exposure to the drugs that cause them. Common drug allergens include certain antibiotics and non-steroidal anti-inflammatory drugs (NSAIDs).

Environmental factors: Avoiding environmental factors such as excessive heat, cold, humidity or direct sunlight can also help minimize hives flare-ups.

3. Lifestyle modifications

Stress reduction: Psychological stress and anxiety may aggravate the symptoms of hives; staying relaxed and reducing stress can help control symptoms. Stress can be relieved through meditation, yoga and deep breathing.

Avoid strenuous exercise: Strenuous exercise may cause an increase in body temperature, which in turn triggers an attack of hives.

Keep cool: Hot baths or exposure to high temperatures may exacerbate the symptoms of hives, and keeping the skin cool can reduce discomfort.

4. Other complementary treatments

Cold compresses: If the skin becomes red, swollen or itchy, cold compresses (e.g. ice packs, cold towels) can be used to relieve symptoms.

Emollients: Keeping the skin moisturized can help reduce itching. Using fragrance-free emollient creams or lotions can relieve dryness and discomfort.

5. Long-term management of chronic urticaria

In patients with chronic urticaria, long-term management may include:

Continued use of antihistamines to control symptoms.

Regular follow-up to assess changes in the condition and adjust the treatment regimen.

For some patients, biologic agents (e.g., omalizumab) or immunosuppressive agents (e.g., cyclosporine, methotrexate) may be treatment options.

6. When to seek medical help

Hives symptoms are recurrent and not easily controlled.

Hives accompanied by difficulty breathing, swelling of the throat, or a generalized allergic reaction (e.g., anaphylaxis) is an emergency and requires immediate medical attention.

Symptoms that last for a long time (e.g., more than 6 weeks, may be chronic hives).

To summarize

Although there may not be a complete cure for urticaria, especially for chronic urticaria, most patients can effectively control their symptoms and avoid frequent recurrence of symptoms through rational medication, avoidance of triggering factors, and good lifestyle habits. If the symptoms persist or worsen, it is recommended to seek medical attention as soon as possible for professional treatment.

**Q10. What is omalizumab, and is it safe?**

Omalizumab, trade name Xolair, is a monoclonal antibody drug that is primarily used to treat allergy-related conditions such as chronic atopic dermatitis (also known as eczema), allergic asthma, and certain types of chronic urticaria (especially in cases where conventional treatments have not been effective).

1. Mechanism of omalizumab

Omalizumab is an anti-immunoglobulin E (IgE) antibody. IgE is an antibody that plays an important role in allergic reactions and is commonly associated with allergic symptoms (e.g. allergic rhinitis, asthma, etc.). By binding to and inhibiting IgE in the body, omalizumab reduces the occurrence of allergic reactions and helps alleviate allergy-related symptoms.

Mechanism of action: Omalizumab reduces the release of allergic mediators (e.g. histamine) and alleviates allergy symptoms by preventing IgE from binding to its receptor.

Indications: It is mainly used for the treatment of patients with chronic urticaria whose symptoms are difficult to be controlled by conventional medications (e.g. antihistamines, steroids, etc.), and patients with allergic asthma.

2. Use of omalizumab

Dosage form and mode of administration: omalizumab is usually administered by subcutaneous injection. For patients with chronic urticaria, monthly injections are usually required.

Course of treatment: During the course of treatment, the doctor will decide on the dosage according to the patient's specific condition and monitor the efficacy and possible side effects regularly.

3. Safety of omalizumab

Omalizumab is a clinically proven biologic that has been approved in many countries for the treatment of allergic diseases such as chronic urticaria. However, like all medications, omalizumab has some side effects and risks, although most patients are able to tolerate them.

Common side effects include:

Injection site reactions: such as redness, pain or swelling.

Allergic reactions: including rash, fever, and shortness of breath. In rare cases, a severe allergic reaction (e.g. anaphylaxis) may be caused, but this is relatively uncommon.

Headache and fatigue: these are commonly reported side effects by some patients.

Respiratory symptoms: such as cough and sore throat.

Less common side effects:

Thrombosis: in rare cases, omalizumab may increase the risk of blood clots.

Immune system abnormalities: there is a very small risk that it may cause an overreaction of the immune system or even trigger autoimmune diseases or blood abnormalities.

Precautions:

History of allergy: you should inform your doctor if you have had an allergic reaction to omalizumab or other ingredients.

Pregnant and lactating women: Omalizumab belongs to Class C drugs (i.e., animal studies have shown that it may be harmful to the fetus, but there have not been enough human studies), so pregnant and lactating women should use it under the supervision of a doctor.

4. Overall safety

Omalizumab has gained extensive experience of use in the treatment of allergic diseases and is safe for use in most patients. However, it is a biologic agent that may trigger individual differences in side effects, so doctors usually monitor patients' health regularly during its use.

If you are considering treatment with omalizumab, it is advisable to discuss its pros and cons in detail with your doctor and have regular follow-up appointments during its use to ensure the safety and efficacy of the treatment.

Conclusion:

Omalizumab has been clinically proven to be effective as a treatment for chronic urticaria and other allergic diseases. It is safe, but some side effects and risks may still exist. As long as it is used under the supervision of a medical professional, it is usually safe and effective in controlling symptoms.

**Q11. Is long-term use of antihistamines safe for infants, young children, and pregnant women? If ineffective, can treatment be escalated to omalizumab as in conventional therapy?**

Antihistamines are used to relieve symptoms caused by allergies, such as allergic rhinitis and hives. Special care needs to be taken regarding the long-term use of antihistamines in infants and pregnant women.

Use of antihistamines in infants and young children

Safety: Some antihistamines (especially first-generation antihistamines such as Diphenhydramine) may cause side effects such as drowsiness and dizziness, which may pose a greater risk to infants and young children, and therefore long-term use is not recommended.

Suggestions for use: If infants and young children do need to use antihistamines, it is recommended that they use them under medical supervision and try to choose second-generation antihistamines (e.g., loratadine, fexofenadine, etc.), which have fewer drowsy effects but still need to be used with caution.

Antihistamines for pregnant women

First-generation antihistamines: Some studies have shown that first-generation antihistamines may pose some risk to pregnant women and fetuses, so they are usually recommended to be avoided by pregnant women.

Second-generation antihistamines: Second-generation antihistamines (e.g., loratadine, fexofenadine, etc.) are relatively safe, but the use of any medication during pregnancy should be supervised by a doctor to weigh the risks against the benefits.

Can I upgrade to omalizumab if my antihistamines are not working well?

Omalizumab is a monoclonal antibody drug used to treat allergic asthma, chronic spontaneous urticaria, and more. Omalizumab is an effective treatment option for patients for whom antihistamines are not effective, especially adult patients suffering from chronic urticaria. However, the following points need to be considered when using omalizumab:

Indications: omalizumab is usually used in adults or children over the age of 12 years, especially in those patients who have not responded well to conventional treatments such as antihistamines. Therefore, the use of omalizumab in infants and pregnant women must be evaluated under the supervision of a physician.

Long-term use: Omalizumab is an injectable drug that is usually used for long-term treatment, but the decision to use it needs to be made by a medical professional on a patient-by-patient basis, especially in special populations such as infants and young children or pregnant women.

Summarize

Infants and children: long-term use of antihistamines is not recommended; if needed, choose a safe drug under the guidance of a doctor.

Pregnant women: avoid the use of first-generation antihistamines, the use of second-generation drugs need to weigh the pros and cons, it is best to use under the advice of a doctor.

When antihistamines are not effective: if conventional treatment is not effective, omalizumab may be a viable option, but it needs to be used under medical supervision, especially for special populations such as pregnant women or infants.

The use of any medication, especially in the infant or pregnant population, should be under the guidance of a medical professional to avoid self-determination.

**Q12. What alternative treatments are available for chronic spontaneous urticaria if omalizumab proves ineffective?**

Chronic Spontaneous Urticaria (CSU) is a common dermatologic condition that manifests as recurrent skin eruptions and itching, and for which a clear cause often cannot be identified. Therapeutically, omalizumab, one of the most commonly used biologics, is effective against IgE-mediated allergic reactions, but not all patients will benefit from it. If Omalizumab is not effective, here are some other treatment options:

1. Antihistamines

FIRST LINE TREATMENT: Antihistamines are the drugs of choice for the treatment of chronic urticaria. Commonly used antihistamines include non-sedating second-generation antihistamines such as Loratadine, Fexofenadine, and Cetirizine.

For patients whose symptoms are not adequately controlled, antihistamines may be used in increments or even in combination with several antihistamines of different mechanisms.

2. Oral steroids

SHORT-TERM USE: In cases of acute exacerbation, oral steroids (e.g., prednisone) may be used to rapidly reduce symptoms. However, long-term use of steroids is not recommended because of the serious side effects (e.g., weight gain, diabetes, hypertension).

3. Immunosuppressants

Cyclosporine A: The immunosuppressant Cyclosporine A (Cyclosporine) may be considered for patients who are not responding well to antihistamines and omalizumab. Cyclosporine A is able to relieve the symptoms of hives by inhibiting T-cell activity.

Meclozine: As an immunomodulatory drug, it can also be used in the treatment of chronic spontaneous urticaria, especially when combined with immune system abnormalities.

4. Biologics

Other biologics such as lebrikizumab: these drugs reduce symptoms by inhibiting specific immune response pathways (e.g., the IL-4/IL-13 pathway), and are indicated for patients in whom omalizumab is ineffective.

Other targeted therapies: For example, biologics targeting IL-5 may be effective in some patients, and although these drugs are more commonly used to treat asthma, there are studies showing potential in certain immune disorders.

5. Chinese medicine

Some patients may respond to Chinese medicine treatment. Commonly used Chinese herbs such as Gui Zhi Tang and Chai Hu Shuo Liver Tang can be individualized based on specific body type and symptoms. Chinese medicine treatment should be carried out under the guidance of a Chinese medicine practitioner.

6. Topical treatment

Topical medications, such as topical antihistamines, analgesic creams, or steroid ointments, can help reduce local itching and redness.

7. Lifestyle modification

Avoidance of triggers: Although chronic urticaria is mostly spontaneous, some patients may be more sensitive to certain factors (e.g. food, drugs, climate, stress, etc.). Avoiding known triggers can be helpful in improving symptoms.

Stress reduction and relaxation: maintaining emotional stability and avoiding excessive stress can help minimize exacerbation of symptoms.

8. Phototherapy

In some resistant cases with persistent symptoms, ultraviolet phototherapy may be helpful in relieving urticaria symptoms, especially in refractory cases.

Taken together, the treatment of chronic spontaneous urticaria needs to be individualized, with treatment strategies adjusted according to the patient's symptoms, response to treatment, and drug resistance. If omalizumab is not effective, further discussion with a dermatologist or immunologist about possible treatment options is recommended to ensure maximum symptom control and improved quality of life.
