# Supplementary material for: Evaluating Generative AI Large Language Models for Urticaria Management: A Comparative Analysis of DeepSeek‐R1 and ChatGPT‐4o
Source: Clin Transl Allergy. 2025 Nov 27;15(11):e70113. doi: 10.1002/clt2.70113 (PMC12658338; doi:10.1002/clt2.70113)
Supplement: Supplementary file 2 — Supporting Information S2 [file CLT2-15-e70113-s003.docx]

**Responses From DeepSeek-R1**

**Q1. What is urticaria?**

Urticaria, commonly known as rubella lumps or hives, is a common skin condition that manifests itself in the form of red or pale-white windy bumps that appear suddenly on the skin, accompanied by intense itching. These masses are of different sizes and irregular shapes, and can appear alone or merge into patches. They usually subside on their own within a few hours, but new masses may appear in other areas.

The pathogenesis of urticaria is mainly related to the release of inflammatory mediators such as histamine, which leads to vasodilatation and increased permeability of the skin blood vessels, thus causing the windburns and itching.

Urticaria can be divided into two types: acute and chronic:

1. acute urticaria: usually lasts less than 6 weeks. Common causes include food allergies (e.g., seafood, nuts), drug reactions (e.g., antibiotics), infections (e.g., viral or bacterial infections), and insect bites.

2. Chronic urticaria: lasting more than 6 weeks, the causes are more complex and may be related to autoimmunity, chronic infections, and physical stimuli (e.g., heat, cold, pressure).

Treatment of urticaria usually involves avoidance of known triggers, use of antihistamines (e.g., cetirizine, loratadine) to control symptoms, and in severe cases, glucocorticosteroids or other immunomodulatory drugs may be needed.

If you have specific symptoms or concerns, it is advisable to consult a dermatologist for a more detailed diagnosis and treatment recommendations.

**Q2. How is urticaria classified?**

Urticaria can be categorized according to its duration, predisposing factors and pathological mechanisms. The following are common classifications:

1. Classification according to duration

Acute urticaria: Duration usually not more than 6 weeks. Common causes include food allergies, drug reactions, infections (e.g. viral or bacterial infections), and insect bites. Symptoms appear suddenly and may subside within a few hours or days.

Chronic urticaria: lasts longer than 6 weeks and may even last for months or years. The cause is more complex and may be related to autoimmunity, chronic infections, physical stimuli (e.g. heat, cold, pressure) and other factors.

2. Classification according to triggers

Spontaneous urticaria: no obvious external triggers, and the windburn and itching appear suddenly. This is the most common type, including acute and chronic spontaneous urticaria.

Induced urticaria: triggered by specific physical or external factors, common types include:

Cold urticaria: caused by cold stimuli, such as cold wind and cold water.

Heat urticaria: caused by a heat stimulus, such as hot water, sunlight, etc.

Solar urticaria: caused by exposure to ultraviolet or visible light.

Stress urticaria: caused by constant pressure on the skin, e.g. tight clothing, prolonged sitting or standing.

Cholinergic urticaria: caused by increased body temperature, e.g. exercise, emotional stress, hot baths, etc.

Contact urticaria: caused by skin contact with certain substances (e.g. latex, plants, animal hair, etc.).

3. Classification according to pathological mechanisms

Immune urticaria: mediated by the immune system, commonly caused by allergic reactions to food, drugs or insect bites.

Non-immune urticaria: does not involve the immune system, may be caused by direct stimuli (such as certain drugs, physical factors).

Autoimmune urticaria: associated with autoantibodies (e.g. anti-IgE or anti-FcεRI antibodies), commonly seen in patients with chronic urticaria.

4. Special types

Angioedema: associated with urticaria but involving deeper skin and mucosal tissues, presenting as localized swelling, commonly found on the eyelids, lips, tongue, and throat.

Hereditary angioedema: a rare hereditary disorder caused by C1 esterase inhibitor deficiency that manifests as recurrent episodes of angioedema.

If you have symptoms of urticaria, it is recommended to seek medical attention as soon as possible, and your doctor will determine the type and develop a treatment plan accordingly.

**Q3. What causes urticaria?**

The pathogenesis of urticaria is complex and involves the immune system, inflammatory mediators and multiple triggers. The following is a detailed description of the pathogenesis and common triggers of urticaria:

I. Pathogenesis

The core mechanism of urticaria is the activation of skin mast cells, which releases histamine and other inflammatory mediators, leading to vasodilatation and increased permeability, thus causing windburn and itching.

1, mast cell activation: mast cells are the key cells in the pathogenesis of urticaria, with high affinity IgE receptors (FcεRI) on their surface. When mast cells are activated, they release inflammatory mediators such as histamine, leukotrienes and prostaglandins.

2, the role of histamine: histamine is the most important inflammatory mediator in urticaria, by binding to the H1 receptor, causing vasodilatation and increased permeability, leading to windburn and itching.

3, immune mechanism: IgE-mediated allergic reaction: certain allergens (such as food, drugs, insect venom) and IgE binding, activating mast cells. Autoimmune response: some patients with chronic urticaria have anti-IgE or anti-FcεRI antibodies, leading to the continued activation of mast cells. Complement system activation: certain infections or immune complexes may activate mast cells through the complement system.

4. Non-immune mechanisms: Certain substances (e.g., aspirin, contrast media) may directly stimulate mast cells to release histamine. Physical stimuli (such as heat and cold, pressure, friction) may also activate mast cells through non-immune pathways.

Second, common triggers

The triggers of urticaria are varied, and the following are some common triggers:

Food: Common allergic foods include seafood, nuts, eggs, milk, chocolate and so on. Food additives (such as preservatives, coloring) may also trigger urticaria.

Medications: Common triggering medications include antibiotics (e.g., penicillin), non-steroidal anti-inflammatory drugs (e.g., aspirin), and anesthetics. Vaccines and biological agents may also cause hives.

Infections: viral (e.g., cold viruses, hepatitis viruses), bacterial (e.g., streptococcus, Helicobacter pylori), and parasitic infections may induce urticaria.

Physical factors: Hot and cold stimuli, sunlight exposure, pressure, friction, vibration and other physical factors may induce urticaria.

Insect bites: Insect bites such as bees, mosquitoes, mites, etc. may cause an allergic reaction leading to hives.

Contact allergens: contact with latex, pollen, animal hair, plant sap, etc. may induce contact urticaria.

Mental factors: stress, anxiety, mood swings may induce or aggravate urticaria.

Systemic diseases: autoimmune diseases (e.g. systemic lupus erythematosus, thyroid disease), tumors, etc. may be associated with chronic urticaria.

Genetic factors: some types of urticaria (such as hereditary angioedema) and genetic mutations.

Third, the classification of urticaria and triggers

According to the triggers and pathogenesis, urticaria can be divided into the following categories:

Spontaneous urticaria: no obvious external triggers, may be related to autoimmunity or chronic infection.

Induced urticaria: triggered by specific physical or external factors, e.g. cold urticaria, heat urticaria, solar urticaria, pressure urticaria, etc.

Special types: cholinergic urticaria: induced by an increase in body temperature (e.g., exercise, emotional excitement).

Contact urticaria: induced by skin contact with certain substances (e.g. latex, plants).

IV. Summary

The pathogenesis of urticaria involves the immune system, mast cell activation and release of inflammatory mediators, with a variety of triggers, including food, drugs, infections, and physical factors. Defining the triggers and avoiding exposure is the key to preventing and treating urticaria. If the symptoms are recurrent or difficult to control, it is recommended to consult a doctor as soon as possible for detailed examination and targeted treatment.

**Q4. How is urticaria diagnosed?**

Diagnosis of urticaria is usually based on history, clinical manifestations and necessary investigations as follows:

1. History taking

Symptom description: find out the pattern of rash, duration, degree of itching and whether it is accompanied by other symptoms (e.g., dyspnea, abdominal pain, etc.).

Triggers inquiry: ask about possible triggers, such as food, drugs, infection, contacts, environmental changes, etc.

Past medical history: to know the history of allergy, family history and chronic diseases.

2. Physical examination

Characteristics of the rash: check for a windswept mass (red or pale white elevation with well-defined borders, accompanied by itching) and whether it subsides within 24 hours.

Other signs: Observe for angioedema (e.g., swelling of eyelids, lips) or other allergic reactions.

3. Laboratory tests

Routine tests: blood counts, C-reactive protein, etc. to assess infection or inflammation.

Allergen testing: e.g. skin prick test or serum-specific IgE test to help identify allergens.

Other tests: depending on the situation, thyroid function and autoantibodies may be performed to rule out other diseases.

4. Trigger test

Under doctor's supervision, observe whether the rash is triggered by contact with suspected triggers (e.g., food, medication) to help identify the triggers.

5. Differential diagnosis

Rule out other similar diseases such as contact dermatitis, drug rash, erythema multiforme.

6. Evaluation of chronic urticaria

If the duration of the disease is more than 6 weeks, further evaluation of the underlying cause, e.g., infection, autoimmune disease, etc., is required.

Summary

The diagnosis of urticaria relies on history and physical examination, supplemented by laboratory tests if necessary. Identification of the causative agent is essential for treatment and prevention of recurrence.

**Q5. Are laboratory tests necessary for diagnosing acute urticaria?**

Acute urticaria usually does not require routine laboratory tests, and the diagnosis relies mainly on history and physical examination. However, your doctor may recommend laboratory tests in the following cases:

1. Suspected infection

If you have symptoms such as fever and sore throat, tests such as routine blood tests and C-reactive protein may be performed to screen for bacterial or viral infections.

2. Suspected allergy

If specific allergens (e.g., food, drugs) are suspected, allergen testing (e.g., skin prick test or serum-specific IgE test) may be performed.

3. Severe symptoms or other abnormalities

If accompanied by dyspnea, abdominal pain, etc., blood tests, liver and renal function tests, etc. may be performed to assess the general condition.

4. Suspected other diseases

If symptoms are atypical or other diseases (e.g. vasculitis, autoimmune diseases) are suspected, related tests (e.g. autoantibodies, complement levels, etc.) may be performed.

Summary

Most acute urticaria does not require laboratory tests, but in cases of severe symptoms, suspicion of infection or allergy, or when the diagnosis is unclear, the doctor may recommend tests to aid in diagnosis and treatment.

**Q6. What are the causes of chronic spontaneous urticaria, and how is it classified?**

Chronic Spontaneous Urticaria (CSU) is a type of urticaria that lasts more than 6 weeks and has a complex cause that is often difficult to pinpoint. The following are its possible causes and classifications:

I. Possible causes of Chronic Spontaneous Urticaria

Autoimmune mechanism: autoantibodies against IgE or IgE receptors are present in some patients, leading to mast cell activation and the release of mediators such as histamine, which triggers urticaria.

Chronic infections: Certain chronic infections (e.g. Helicobacter pylori, viral hepatitis, sinusitis, etc.) may induce or aggravate urticaria.

Chronic inflammation: chronic inflammatory diseases (e.g. thyroiditis, systemic lupus erythematosus, etc.) may be associated with urticaria.

Drugs or foods: Certain drugs (e.g., NSAIDs, ACEIs) or foods (e.g., additives, preservatives) may induce or exacerbate symptoms.

Physical factors: some patients are sensitive to physical stimuli such as cold, heat and pressure, which may induce urticaria.

Other factors: mental stress, endocrine disorders, etc. may also affect the condition.

Classification of chronic spontaneous urticaria

Classification according to the cause

Autoimmune urticaria: related to autoantibodies, patients may have other autoimmune diseases.

Infection-related urticaria: related to chronic infection, symptoms may be relieved after treatment of infection.

Idiopathic urticaria: the cause is unknown and accounts for the majority of cases.

Classification according to course

Persistent urticaria: symptoms persist without significant periods of remission.

Intermittent urticaria: Symptoms recur with periods of remission in between.

Classification according to symptom severity

Mild: symptoms are mild and have little impact on life.

Moderate: Symptoms are more obvious and affect daily life.

Severe: severe symptoms, accompanied by angioedema or other systemic symptoms, significantly affecting life.

Summary

The etiology of chronic spontaneous urticaria is complex and may be associated with a variety of factors including autoimmunity, infection, and medications. Depending on the cause, duration and severity of the disease, it can be classified to help develop a personalized treatment plan.

**Q7. How is the severity and activity of urticaria assessed?**

The severity or activity of urticaria is determined primarily on the basis of the frequency and duration of symptoms, the extent of the rash, and its impact on quality of life. Commonly used tools include clinical assessment and standardized rating scales.

I. CLINICAL ASSESSMENT

Extent of rash

Mild: rash is limited and small in extent.

Moderate: Rash is extensive and involves multiple sites.

Severe: Rash is extensive and generalized.

Degree of itching

Mild: itching is mild and does not affect daily life.

Moderate: Itching is obvious and affects some daily activities.

Severe: severe itching, seriously affecting life.

Frequency of attacks

Mild: less than 1 attack per week.

Moderate: several episodes per week.

Severe: daily or persistent.

Concomitant symptoms

Mild: no or mild angioedema.

Moderate: with mild angioedema.

Severe: with severe angioedema or systemic symptoms (e.g. dyspnea, abdominal pain, etc.).

Standardized rating scale

1. Urticaria Activity Score (UAS): assesses the number of windsocks and degree of itching, recorded daily, with a total score of 0-6:

Number of windsocks: 0=none, 1=1-6, 2=7-12, 3=more than 12.

Degree of itching: 0=none, 1=mild, 2=moderate, 3=severe.

2, Urticaria Control Test (UCT)

Assesses control of urticaria over the past 4 weeks with a total score of 0-16: 16 indicates complete control and ≤11 indicates poor control.

3、Quality of life score: Use tools such as the Dermatologic Quality of Life Index (DLQI) to assess the impact of urticaria on the patient's life.

3、Comprehensive assessment

Mild: mild symptoms, low frequency of flare-ups, little impact on life.

Moderate: more obvious symptoms, higher frequency of attacks, some impact on life.

Severe: severe symptoms, frequent or persistent attacks, significant impact on life, may be accompanied by systemic symptoms.

Summary

The severity of urticaria is assessed by a combination of rash extent, itchiness, frequency of flare-ups, and concomitant symptoms, and standardized rating scales (e.g., UAS, UCT) can help quantify activity and control.

**Q8. Is urticaria curable?**

The likelihood of cure for urticaria varies depending on the type and cause, here are the specifics:

I. Acute urticaria

Likelihood of cure: most can be cured.

Treatment: Remove triggers (e.g., food, drugs, infections) and use antihistamines, usually within a few days to weeks.

II. Chronic spontaneous urticaria (CSU)

Likelihood of cure: some patients can be cured, some need long-term management.

Treatment: antihistamines are preferred, and omalizumab and cyclosporine can be used when the effect is poor. Some of the patients' symptoms will be relieved by themselves after several years.

Third, physical urticaria

Possibility of cure: difficult to completely cure, but can be controlled.

Treatment: avoid triggers (such as cold, heat, pressure), use antihistamines to control symptoms.

IV. Other types of urticaria

Possibility of cure: depends on the cause.

Treatment: e.g. cholinergic urticaria controlled by avoidance of sweating and antihistamines, contact urticaria by avoidance of allergens.

V. LONG-TERM MANAGEMENT

LIFESTYLE ADJUSTMENT: Avoid triggers, reduce stress, and maintain healthy lifestyle habits.

Regular follow-up: Chronic patients need regular follow-up to adjust the treatment program.

Summarize

Acute urticaria is usually curable, chronic urticaria can be cured or relieved on its own in some patients, and some need long-term management. Through standardized treatment and lifestyle adjustments, most patients can effectively control their symptoms.

**Q9. How is urticaria treated?**

The goal of treatment for hives is to relieve symptoms and minimize flare-ups, with specific options varying by type and severity. The following are common treatment methods:

First, acute urticaria treatment

1, remove triggers: identify and avoid allergens (such as food, drugs, infections, etc.).

2, antihistamines:

First-line treatment: second-generation antihistamines (such as cetirizine, loratadine), once a day, with few side effects.

The effect is not good: can increase the dose or joint use of other antihistamines.

3、Glucocorticoids: short-term use: for severe cases, oral prednisone for 3-5 days, avoid long-term use.

4, epinephrine: emergency: for anaphylaxis or severe angioedema, immediate intramuscular injection of epinephrine.

Second, chronic spontaneous urticaria (CSU) treatment

1, antihistamines: first-line treatment: second-generation antihistamines, can be increased to four times the dose when the conventional dose is ineffective.

2, omalizumab: second-line treatment: anti-IgE monoclonal antibody, used in patients with moderate to severe disease who are ineffective on antihistamines, monthly subcutaneous injection.

3、Immunosuppressants: third-line treatment: e.g., cyclosporine, used for patients with ineffective omalizumab, need to monitor side effects.

4, other drugs: leukotriene receptor antagonist: such as montelukast, can be combined with antihistamines.

Third, physical urticaria treatment

1, avoid triggers: such as cold, heat, pressure and other physical stimuli.

2, antihistamines: routine use, prevention and control of symptoms.

Fourth, other types of urticaria treatment

1, cholinergic urticaria: avoid sweating, use antihistamines.

2、Contact urticaria: avoid contact with allergens, use antihistamines.

V. Long-term management

1. Lifestyle adjustment: avoid triggers, reduce stress, and maintain healthy living habits.

2、Regular follow-up: chronic patients need regular follow-up and adjustment of treatment program.

Summary

Treatment of urticaria includes removal of triggers, antihistamines, glucocorticoids, etc. Chronic patients may need omalizumab or immunosuppressants. Through standardized treatment and lifestyle modification, most patients can effectively control their symptoms.

**Q10. What is omalizumab, and is it safe?**

Omalizumab (Omalizumab) is a biologic agent used primarily for the treatment of moderate to severe allergic asthma and chronic idiopathic urticaria. It relieves symptoms by inhibiting the action of immunoglobulin E (IgE) and reducing allergic reactions and inflammation.

Safety - Omalizumab has demonstrated a high level of safety in clinical trials and in real-world applications, but some potential side effects remain:

Common side effects: injection site reactions (e.g., redness, swelling, pain). Headache. Upper respiratory tract infection

Rare but serious side effects: allergic reactions (e.g., hives, dyspnea). Cardiovascular events (e.g., myocardial infarction, stroke)

Increased risk of parasitic infections

Precautions

HISTORY OF ALLERGY: Patients with a history of hypersensitivity to omalizumab or its components should avoid use.

Monitoring: Periodic monitoring for signs of allergic reactions and infection is required during treatment.

Special Populations: Pregnant and lactating women and children should consult a physician before use.

Conclusion

Omalizumab is safe and effective in suitable patients, but it should be used under medical supervision and closely monitored for side effects.

If you have further questions, it is recommended to consult a medical professional.

**Q11. Is long-term use of antihistamines safe for infants, young children, and pregnant** **women? If ineffective, can treatment be escalated to omalizumab as in conventional therapy?**

Caution is needed in the use of antihistamines in infants and pregnant women, and long-term use should be under medical supervision. For poor results, upgrading to biologics such as omalizumab should also be done under strict medical advice.

1. Use of antihistamines in infants and young children

Short-term use: usually safe, but dose needs to be adjusted according to weight and age.

Long-term use: requires a physician's assessment of potential risks, such as developmental effects. 2.

2. Antihistamines for pregnant women

Short-term use: Some drugs such as loratadine and cetirizine are relatively safe during pregnancy.

Long-term use: doctor's assessment is needed to avoid drugs that may affect the fetus.

3. Escalation therapy when not effective

Omalizumab: indicated for moderate to severe allergic asthma and chronic urticaria, but safety data in infants, children and pregnant women are limited and require rigorous evaluation by a doctor.

4. Precautions

Consult your doctor: You should consult your doctor before using any medication to ensure safety and efficacy.

Regular assessment: Long-term use of medication requires regular monitoring and timely adjustment of the treatment regimen.

In conclusion, the use of antihistamines or upgrading to omalizumab in infants, children and pregnant women should be done under medical supervision to ensure safety and efficacy.

**Q12. What alternative treatments are available for chronic spontaneous urticaria if omalizumab proves ineffective?**

If omalizumab does not work well for chronic spontaneous urticaria (CSU), there are other treatment options. Here are some common alternative or complementary treatment options:

1. other biologics

Ligelizumab: a new anti-IgE monoclonal antibody that may be effective in some patients.

Other targeted therapies: such as anti-IL-5, anti-IL-4/IL-13 and other biologics are currently under investigation and may be suitable for specific patients.

2. Immunosuppressants

Cyclosporine (Cyclosporine): indicated for refractory CSU, but close monitoring of side effects such as renal function and blood pressure is needed.

Methotrexate (Methotrexate): can be used in some patients, but regular monitoring of liver function and blood picture is needed.

3. Anti-inflammatory drugs

Glucocorticoids: short-term use can control acute symptoms, but long-term use has more side effects and requires caution.

Leukotriene receptor antagonists (e.g. montelukast): can be used as an adjunctive therapy, especially for patients with asthma or allergic rhinitis.

4. Other antihistamines

High-dose antihistamines: when the standard dose is ineffective, an increase in dose (up to 4 times the standard dose) may be attempted, subject to medical supervision.

Different types of antihistamines: e.g. H2 receptor antagonists (e.g. ranitidine) can be used as complementary therapy.

5. Phototherapy

Ultraviolet light therapy (UVB/UVA): may be effective in some patients, but evidence is limited.

6. Other experimental treatments

Autologous serum therapy (ASST): for some patients with autoimmune urticaria.

Plasma exchange: may be considered in rare severe cases.

7. Lifestyle modifications

Avoidance of triggers: e.g. physical factors such as stress, heat, cold, friction.

Dietary modifications: some patients may be sensitive to specific foods; exclusion diets may be attempted.

8. Psychological support

Psychotherapy: Chronic urticaria may be psychologically burdensome to the patient; psychological support and cognitive behavioral therapy (CBT) may help to improve quality of life.

Precautions

Individualized treatment: The treatment plan should be tailored to the patient's specific situation and needs to be carried out under the supervision of a doctor.

Regular follow-up: The efficacy and side effects need to be assessed regularly during treatment, and the regimen should be adjusted in a timely manner.

If omalizumab is not effective, it is recommended to discuss other treatment options with your doctor to find the most suitable individualized treatment strategy.
